# COVID-19 *in-vitro* Diagnostics: State-of-the-Art and Challenges for Rapid, Scalable, and High-Accuracy Screening

**DOI:** 10.3389/fbioe.2020.605702

**Published:** 2021-01-28

**Authors:** Zeina Habli, Sahera Saleh, Hassan Zaraket, Massoud L. Khraiche

**Affiliations:** ^1^Neural Engineering and Nanobiosensors Group, Biomedical Engineering Program, Maroun Semaan Faculty of Engineering and Architecture, American University of Beirut, Beirut, Lebanon; ^2^Department of Experimental Pathology, Immunology and Microbiology, Faculty for Medicine, American University of Beirut, Beirut, Lebanon; ^3^Center for Infectious Diseases Research, Faculty of Medicine, Beirut, Lebanon

**Keywords:** SARS-CoV-2, COVID-19, pandemic, diagnostics, screening, serological immunoassays, point-of-care

## Abstract

The world continues to grapple with the devastating effects of the current COVID-19 pandemic. The highly contagious nature of this respiratory disease challenges advanced viral diagnostic technologies for rapid, scalable, affordable, and high accuracy testing. Molecular assays have been the gold standard for direct detection of the presence of the viral RNA in suspected individuals, while immunoassays have been used in the surveillance of individuals by detecting antibodies against SARS-CoV-2. Unlike molecular testing, immunoassays are indirect testing of the viral infection. More than 140 diagnostic assays have been developed as of this date and have received the Food and Drug Administration (FDA) emergency use authorization (EUA). Given the differences in assasy format and/or design as well as the lack of rigorous verification studies, the performance and accuracy of these testing modalities remain unclear. In this review, we aim to carefully examine commercialized and FDA approved molecular-based and serology-based diagnostic assays, analyze their performance characteristics and shed the light on their utility and limitations in dealing with the COVID-19 global public health crisis.

## Introduction

Severe acute respiratory syndrome coronavirus 2 (SARS-CoV-2) leads to the infectious disease COVID-19, which was first reported in Wuhan, China in December 2019. The disease has since spread across the globe infecting over 215 countries. SARS-CoV-2 is highly contagious and spreads from person-to-person by the respiratory route, primarily via droplets, aerosols, and contact with contaminated surfaces and fomites (Yeasmin et al., 2020). Its reproduction number (R_0_) is estimated to be around 2.68 with a doubling time of 6.4 days. On the other hand, its incubation period from infection to first symptoms is on average 5 days with a possibility of reaching 14 days (Wu et al., 2020b). The clinical manifestations of COVID-19 include fever, cough, fatigue, and breathing difficulties, which could result in severe complications, such as severe pneumonia and respiratory distress syndrome (ARDS). To date, only a very limited number of therapeutic options have shown little effects on reducing COVID-19 associated death, such as remdesivir and dexamethasone (reviewed in Kaddoura et al., 2020).

In the absence of proven antiviral drug therapies and commercially available vaccines, the current pandemic containment and mitigation strategy is relying on the isolation of the infected individuals and their close contacts in addition to social distancing. The problem is exacerbated by the presence of many asymptomatic patients that can only be identified via molecular based disease screening methods. Due to that, mass testing has been central to the worldwide disease containment efforts which represented a challenge to cost, scalability, and speed of state-of-the-art viral screening technology.

Real-time reverse transcriptase-polymerase chain reaction (RT-PCR) is the clinical gold standard for the etiological diagnosis of COVID-19 in which viral RNA is directly detected in respiratory specimens by means of molecular biology techniques. On the other hand, serology-based immunoassays are being used in central labs or rapid testing for epidemiological surveillance to detect antibodies produced by infected individuals in response to SARS-CoV-2 exposure. So far, scientists are racing to develop novel approaches for rapid testing with high sensitivity and low cost to meet the diagnostic challenges. This goes hand in hand with the evolving effort to battle COVID-19, as many countries have opted to employ safe reopening to mitigate the economic crisis and manage health consequences. Safe reopening measures include investments in the public health infrastructure, implementing rapid and sensitive diagnostics of suspected cases, contact tracing and integrating social distancing and quarantining when needed (Gottlieb et al., 2020).

In this body of work, we carry out a deep technical analysis of the current COVID-19 diagnostic tests approved by the FDA for emergency use in Clinical Laboratory Improvement Amendments (CLIA)-certified molecular diagnostics laboratories. The work also closely probes each testing technology to identify their advantages and disadvantages and the clinical and field challenges still facing mass testing for COVID-19.

## SARS-CoV-2 Virology and Pathophysiology

### SARS-CoV-2 Viral Structure and Genome

**Figure 1:**
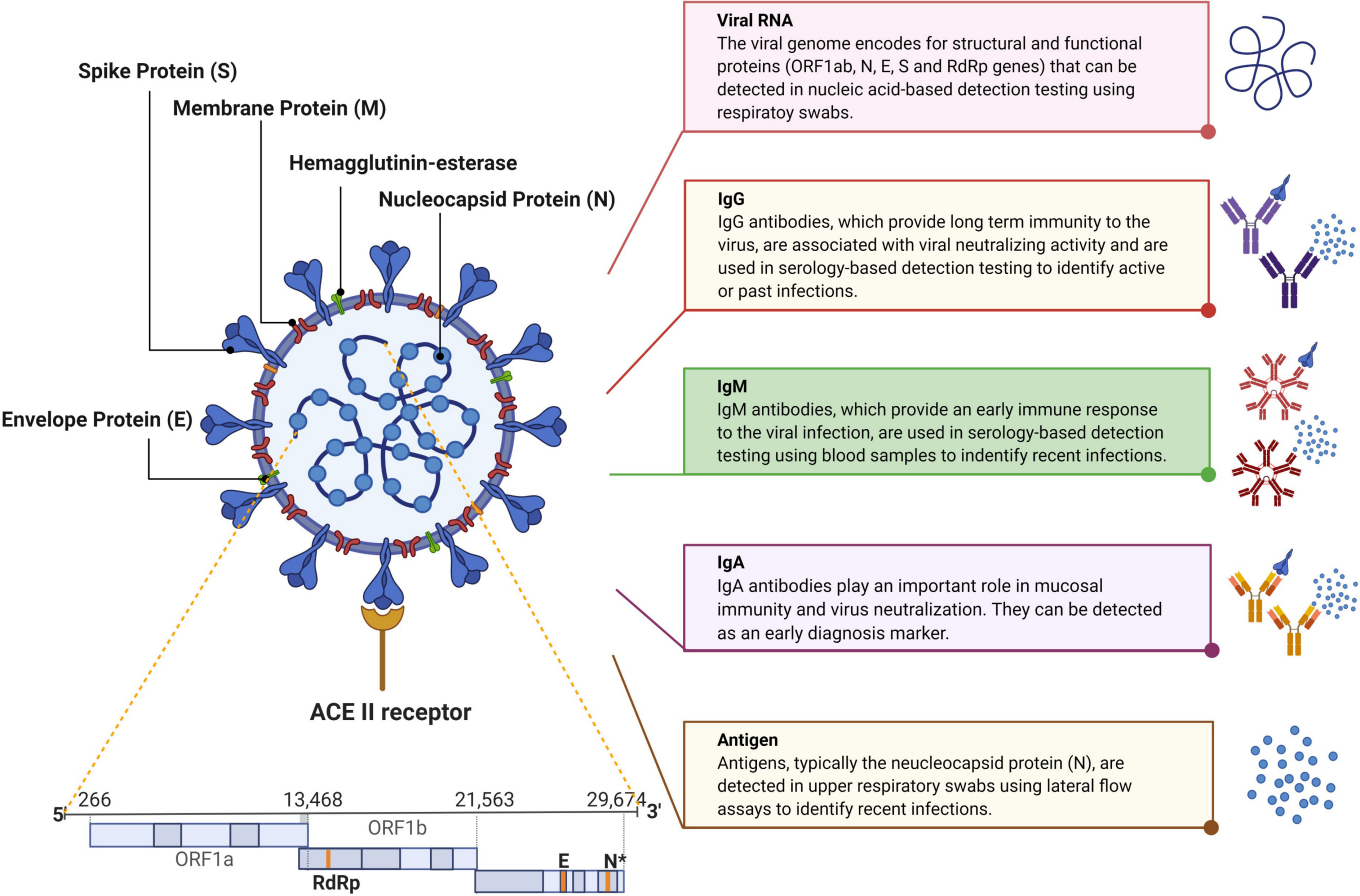
SARS-CoV-2 structure, genome, and diagnostic targets.

SARS-CoV-2 is an enveloped large positive-sense single-stranded RNA virus with a genome of ~30 kb (Figure 1). The virus belongs to the genus *betacoronavirus* and has a diameter of 50–200 nm. SARS-CoV-2 is characterized by the spike glycoproteins protruding from its surface giving it the characteristic crown-like appearance under the electron microscope (Cascella et al., 2020; Zhou et al., 2020). The SARS-CoV-2 genome encodes for four major structural and functional proteins: the spike (S), membrane (M), envelope (E), and nucleocapsid (N) proteins (Zou et al., 2020). The S protein consists of two functional subunits, S1 and S2; S1 is responsible for recognizing and binding the host cell receptor, angiotensin-converting enzyme 2 (ACE2), while S2 mediates membrane fusion. The M protein is the most abundant structural protein that defines the shape of the virus. The N protein is the most abundantly shed viral protein during infection and can be detected in serum and urine samples within the first 2 weeks of infection. The smallest major structural protein, E protein, participates in viral assembly and pathogenesis (Wang et al., 2020).

### SARS-CoV-2 Pathophysiology

Several studies have shown that SARS-CoV-2 infects cells with high expression of ACE2 receptors, such as type II pneumocytes in the upper and lower respiratory system, endothelium, myocardium, and gastrointestinal mucosa cells (Astuti and Ysrafil, 2020; Bobeck et al., 2020; Hoffmann et al., 2020). The virus enters the cell via endoplasmic uptake. Following fusion with the endosomal membrane, the viral RNA genome is released into the cytoplasm where replication of the virus genomes and production of its proteins take place. The newly synthesized nucleocapsid, made up of viral RNA and N protein, are imported into the endoplasmic reticulum-Golgi intermediate compartment (ERGIC) and the viral proteins are assembled with the rest of the proteins to form progeny viruses. The latter are then transferred in vesicles to the plasma membrane where they exit (Fehr and Perlman, 2015).

The clinical manifestations of COVID-19 patients range from mild (e.g., fever, non-productive cough, dyspnea, fatigue, sore throat, etc.) to severe complications, such as pneumonia and ARDS and may lead to death in chronic severe cases. Besides, infected individuals show high production of leukocytes, lymphopenia, and elevated levels of cytokines (e.g., IL-6) (Liu et al., 2020; Rothan and Byrareddy, 2020). The immune response triggered against this virus involves the secretion of three types of immunoglobulins, anti-SARS-CoV-2 immunoglobulins M (IgM), anti-SARS-CoV-2 immunoglobulins G (IgG), and anti-SARS-CoV-2 immunoglobulins A (IgA), which are essential biomarkers for identifying individuals affected by COVID-19, including those who may be asymptomatic or have recovered (Jacofsky et al., 2020).

## SARS-CoV-2 Biomarkers for Diagnosis

**Figure 2:**
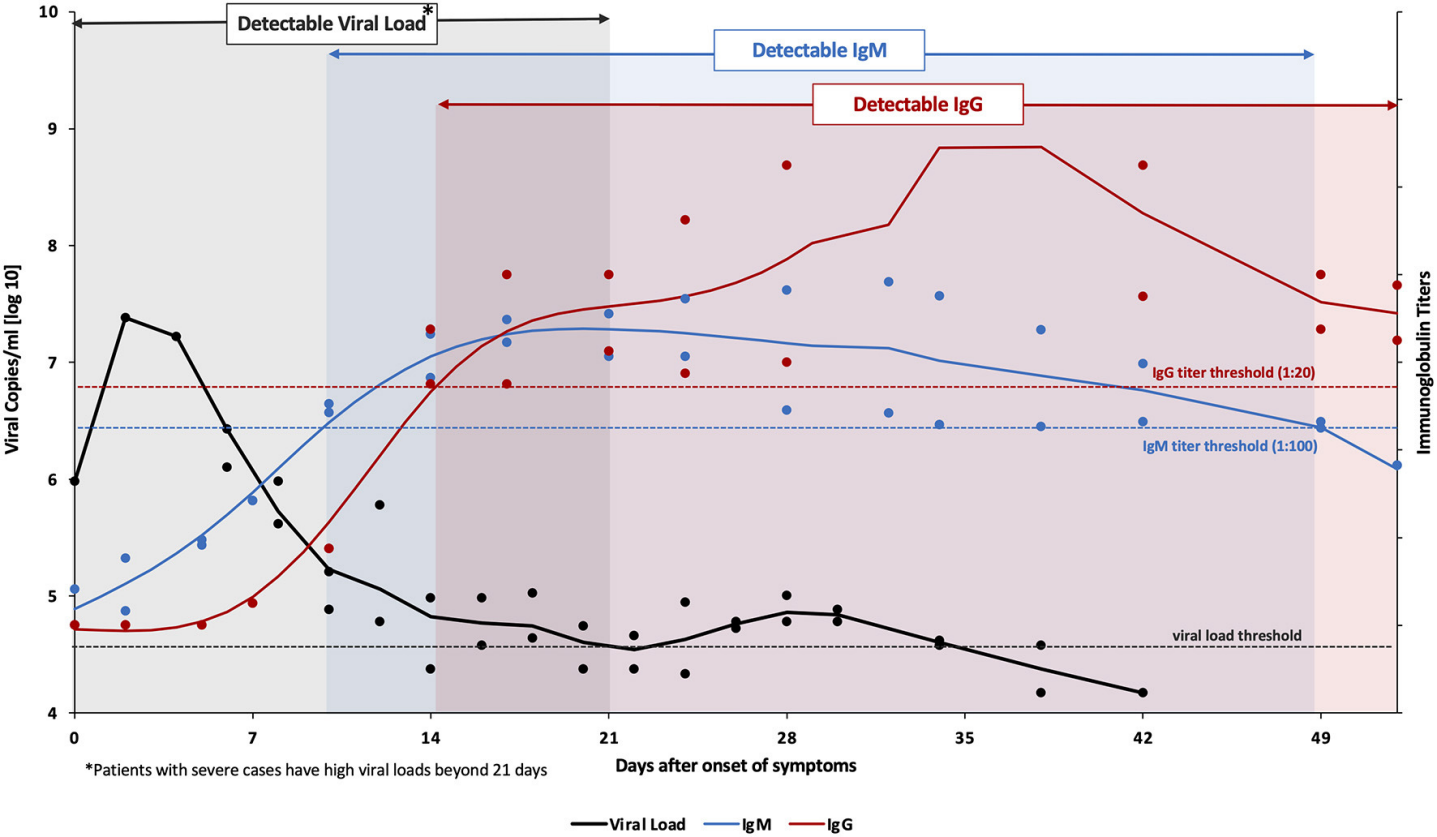
Variation levels of viral load, IgM and IgG post SARS-CoV-2 infection. Data collected from Tan et al. from 67 confirmed COVID-19 patients (both mild and severe cases) (Tan et al., 2020). Viral RNA (amplified ORF1ab gene) was detected in nasopharyngeal swabs using the Qiamp® viral RNA mini kit (QIAGEN, Hilden, Germany). The viral load threshold (black dashed line) indicates the detection limit of this kit corresponding to a cycle threshold value of 38 which is equivalent to 104.577 genomic copies/mL. On the other hand, IgM & IgG titers (anti-SARS-CoV-2 nucleocapsid immunoglobulins) were analyzed in serum samples using ELISA kits (Livzon Diagnostics Inc., Zhuhai, China). The blue and red dashed lines denote the cutoff value for a positive result. The bold lines represent the trends, fitted using smoothing splines in Matlab.

Current nucleic acid-based diagnostic assays target the E, S, RNA-dependent RNA polymerase (RdRp), N, and/or open reading frame (ORF1ab) genes (Chan et al., 2020; Chu et al., 2020; Corman et al., 2020). For these assays, upper and lower respiratory tracts samples are collected via nasopharyngeal (NP) or oropharyngeal (OP) swabs, sputum, endotracheal aspirate, bronchoalveolar lavage, or saliva where the SARS-CoV-2 RNA was found to be higher than in other samples, such as blood and stool. The latter two samples are only collected for research purposes (Vogels et al., 2020a; Zou et al., 2020). The analytical sensitivity of such assays is affected by the temporal profile of the viral load over the course of infection. Several studies suggest that the viral load peaks shortly around the time or even before symptoms onset then decreases quickly and monotonically within the first 7 days (Becherer et al., 2020; To et al., 2020b). Additionally, the virus remains detectable for 20 days or longer after symptom onset (Figure 2) (Zou et al., 2020). This means that false negatives may possibly be obtained early and late stages of the infection when using molecular diagnostic assays for testing. In addition, given the relatively short detection window of these assays, the true extent of exposure to the virus in a population may be underestimated.

Serology-based immunoassays have also been developed to detect antibodies for SARS-CoV-2 in the serum or plasma of patients, namely anti-S and anti-N IgM, IgG, and IgA (To et al., 2020a; Zou et al., 2020). IgM provides the first line of defense during a viral infection. Also, the IgM response to SARS-CoV-2 was reported to occur earlier than other immunoglobulins, at around 10 days after infection, but then decreases rapidly after 35 days and disappears (Figure 2). IgG on the other hand provides long-term immunity and immunological memory and is detectable starting 13–21 days after infection and persists for a longer time (Figure 2) (Tan et al., 2020). On the other hand, IgA levels in the blood and saliva specimens have been correlated with COVID-19 severity; therefore it can be used as a complementary biological marker for COVID-19 identification (Ma et al., 2020). Thus, antibodies indicate the true exposure to the virus in an individual since they can be detected long after the virus has disappeared (Demey et al., 2020). However, the lack of detectable antibodies at the early stages of the infection makes serology-based immunoassays unsuitable for the diagnosis of an active infection.

Although nucleic acid assays are rather vital for diagnostic purposes, especially in cases of mild to acute infection, serology-based testing is proving to be increasingly important in understanding the dynamics of the current pandemic as it continues to spread. Complementary to molecular-based assays and immunoassays, the FDA recently approved tests that can detect the viral antigens, mainly the N protein. Similar to the viral load, SARS-CoV-2 proteins are maximal during the first week of symptoms onset, and rapidly decline during the recovery phase. The diagnostic targets are highlighted in Figure 1 whereas the variation in the levels of SARS-CoV-2 viral load, IgM, and IgG post-infection are shown in Figure 2.

## Molecular-Based Assays for SARS-CoV-2 Nucleic Acid Detection

**Figure 3:**
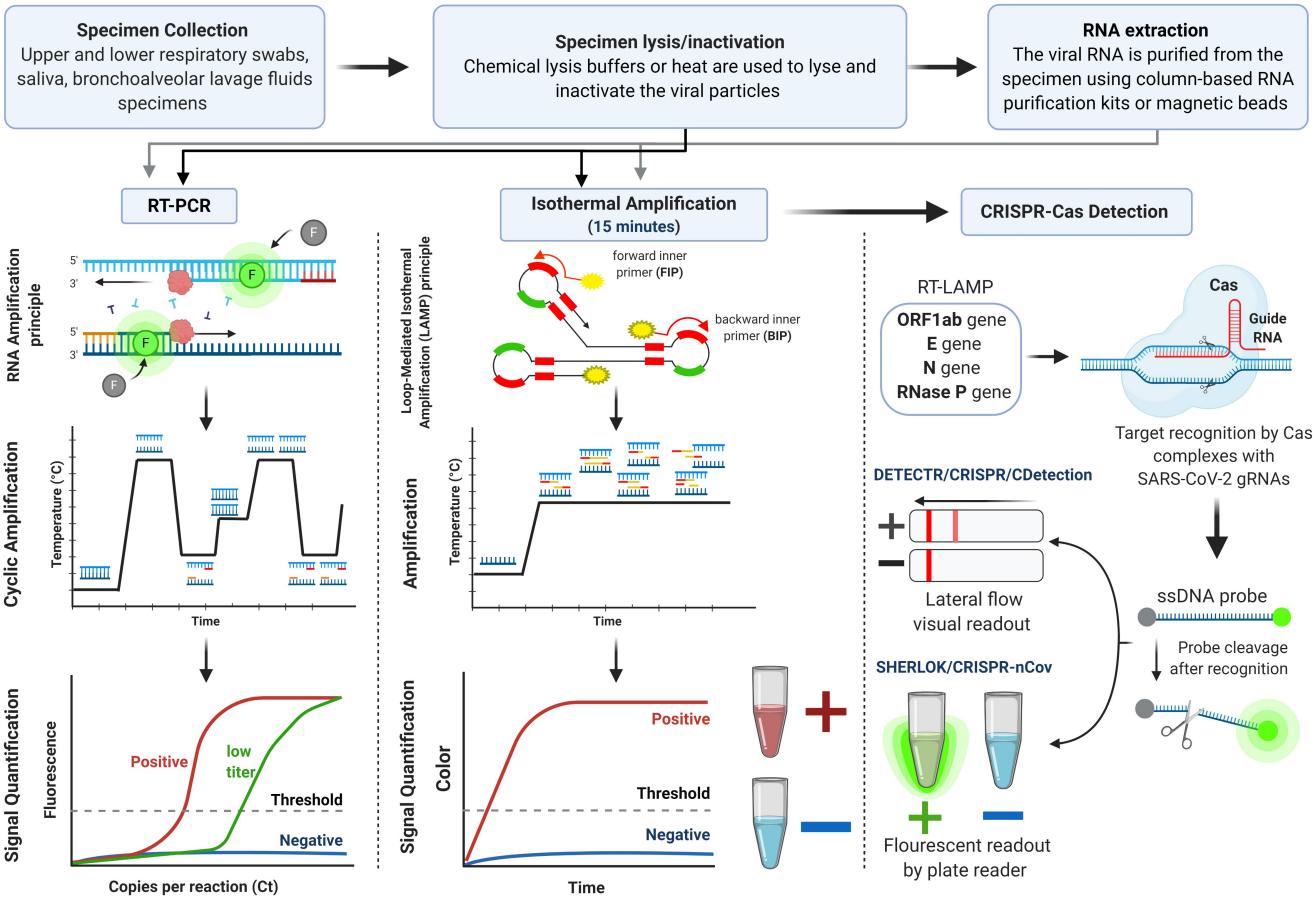
Workflow for nucleic acid-based detection assays.

As soon as the genome sequence of SARS-CoV-2 was made publicly available on the Global Initiative on Sharing All Influenza Data (GISAID) (Udugama et al., 2020; Wu et al., 2020a), numerous molecular COVID-19 diagnostic kits were developed. This section is an analysis of the clinically conventional and novel methodologies for SARS-CoV-2 nucleic acid detection that received FDA EUA and/or European Conformity (CE Marking). The molecular detection kits are outlined in Supplementary Table 1 [data obtained from (FDA, 2020b) and (FINDdx, 2020)], and their workflow is illustrated in Figure 3.

### Reverse Real-Time Polymerase Chain Reaction (RT-PCR) Assays for SARS-CoV-2 Detection

The current clinical standard molecular test for SARS-CoV-2 nucleic acid detection is the quantitative reverse RT-PCR assay. These assays were the first developed for COVID-19 diagnosis and remain the most used assays. More than one hundred RT-PCR kits have been designed and prototyped and have received FDA EUA approval for COVID-19 diagnosis genetically (presented in Supplementary Table 1). RT-PCR reaction involves the reverse transcription of SARS-CoV-2 genomic RNA into a complementary DNA (cDNA) followed by amplification of targeted sequences using a set of specific primers. A variety of gene targets with comparable sensitives are used to recognize regions of the viral genes, such as structural proteins genes (N, E, S, M genes) and confirmatory genes (ORF1ab and RdRp genes). The latter genes are used to avoid potential cross-reactivity with other coronaviruses and any possibility of genetic drift in SARS-CoV-2. The Centers for Disease Control and Prevention (CDC) recommends screening with nucleocapsid protein targets (N1 and N2) while the World Health Organization (WHO) recommends targeting the E gene followed by the confirmatory RdRp gene (Tang et al., 2020). The viral RNA is measured by the cycle threshold (Ct), which is the number of amplification cycles needed to produce a measurable fluorescent signal. The lower the Ct values, the higher the viral RNA load in a sample. The intensity of the fluorescent signal reflects the momentary amount of DNA amplicons; after 35–40 amplification cycles, the viral DNA is quantified and can be detected even if the starting viral RNA amount is small (reviewed in Kralik and Ricchi, 2017). The test typically takes 6–8 h on average, but with the need for clinical sample collection and transfer, the test requires 24 h at best. A positive PCR result reflects the presence of the viral RNA but does not necessarily indicate the presence of infectious viruses within the sample. In addition, PCR positivity is specimen dependent; it declines more slowly in sputum compared to fast declination in NP swabs (Wölfel et al., 2020).

Although RT-PCR is the gold standard with high specificity (~100%), sensitivity, and accuracy, the procedure is labor-intensive and relies on sophisticated instrumentation usually located at central laboratories and requires the use of biosafety level 2 cabinets (Younes et al., 2020). These assays are time-consuming and require technical expertise in specialized and controlled space making them unsuitable for deployment as point-of-care rapid diagnostics but have the advantage of being high throughput.

To overcome the limitations of conventional real-time RT-PCR assays, fully automated high-quality molecular point-of-care diagnostic platforms have been developed. For example, Roche Molecular Systems, Inc. developed the automated Cobas® SARS-CoV-2 RT-PCR test which is intended for qualitative detection of the viral nucleic acid in NP and OP swabs. Roche Cobas® can deliver results within 3.5 h while running up to 96 tests simultaneously. Also, depending on the instrumentation used, it can process 384 specimens with the cobas 6800 System or 1,056 specimens with the cobas 8800 System in <8 h. Additionally, the Xpert® Xpress SARS-CoV-2 test developed by Cepheid (California, USA) integrates nucleic acid extraction, transcription and amplification in a single cartridge, and provides results within 45 min with 100% sensitivity and 99% specificity-agreement when compared to Roche Cobas® SARS-CoV-2 tests. Another forerunner in the field of testing is the BioFire Diagnostics, LLC, which developed the multiplexed BioFire Respiratory Panel 2.1 (RP2.1) closed system. The limit of detection (LoD) of the BioFire is 300 viral genomic copies/mL and gives results within 45 min as well.

### Reverse Transcription Loop-Mediated Isothermal Amplification (RT-LAMP) Assays for SARS-CoV-2 Detection

The limitations of RT-PCR, namely the complex and expensive instrumentation needed for thermal cycling, led to the development of isothermal nucleic acid amplification tests that overcome these issues (Huang et al., 2020). Currently, these point-of-care tests are becoming more appealing in clinical applications, mainly due to fast processing times and low-cost devices needed (Kashir and Yaqinuddin, 2020). They obviate the need for trained personnel, access to expensive laboratory equipment and high-tech facilities for sample processing, which is particularly important in developing countries with limited access to such resources. The most prominently used isothermal nucleic acid amplification test is the loop-mediated isothermal amplification (LAMP) (Nguyen et al., 2020). It was first described by Notomi et al. (in review), then further optimized for accelerated amplification by Nagamine et al. (Becherer et al., 2020). This technique uses four to six primers, inner and outer, which recognize six to eight different regions in the target DNA sequence, and a DNA polymerase with strand-displacement activity. The process is carried out at a constant temperature ranging between 60 and 65°C, synthesizing target DNA up to 10^9^ copies in less than an hour (Nguyen et al., 2020). The final products of LAMP are multiple inverted repeats of the target DNA with stem-loop structure (Becherer et al., 2020). LAMP combined with reverse transcription (RT-LAMP) allows for the direct detection of RNA and can thus be used for the rapid detection of SARS-CoV-2 (Kashir and Yaqinuddin, 2020; Park et al., 2020). Colorimetric or fluorescent read-out could be used for the visualization of the results (Becherer et al., 2020). This assay achieves a sample-to-result time of 1–2 h. This gives RT-LAMP a great advantage over RT-PCR that has a sample-to-result time of 6–8 h, ensuring a rapid response required in massive virus outbreaks. The diagnostic sensitivity and specificity of RT-LAMP assays designed for the detection of SARS-CoV-2 were found to be comparable to those of RT-PCR in various studies (Butt et al., 2020; Zhang et al., 2020b). These features suggest that RT-LAMP can be employed in the diagnosis of COVID-19 with high levels of precision, low levels of background signal and more tolerance to PCR-inhibitors (Becherer et al., 2020).

A few isothermal nucleic acid amplification assays have received FDA EUA approval and are currently available, including the Abbott ID NOW COVID-19 assay that utilizes isothermal amplification to detect the RdRp gene of SARS-CoV-2 RNA (Basu et al., 2020). This assay provides positive results within 5 min and negative results within 13 min with a LoD of 125 genome equivalents/mL and reported 100% sensitivity and specificity as claimed by its manufacturer (Harrington et al., 2020). However, on May 15, 2020, the FDA issued a public warning about Abbott ID NOW COVID-19 accuracy and performance. The test has reported negative results in one third of the samples tested positive by Cepheid Xpert Xpress when using NP swabs and in 45% of positive samples using dry nasal swab samples (Basu et al., 2020). Another assay that employs isothermal nucleic acid amplification is the iAMP® COVID-19 Detection Kit developed by Atila Biosystems Inc. which targets ORF1ab and N genes of SARS-CoV-2 RNA (Bulterys et al., 2020). It received FDA EUA in April 2020. The sample-to-result time is around 1 h and up to 94 samples per instrument can be run with a LoD of 4,000 copies/mL. The manufacturers reported 98% sensitivity and 93% specificity.

### CRISPR-Based Assays for SARS-CoV-2 Detection

Over the past two years, the use of CRISPR in infectious diseases diagnostics has been gaining momentum (Bhattacharyya et al., 2018). CRISPR belongs to a family of palindromic nucleic acid repeats, found in bacteria, that can be recognized and cut by a unique set of effector enzymes known as the CRISPR-associated (Cas) proteins. The Cas enzymes display exceptionally sensitive and specific nucleic acid detection modalities as they can be programmed to identify and cut SARS-CoV-2 RNA sequences.

Mammoth Biosciences and Sherlock Biosciences have reconfigured their CRISPR-based platforms independently to rapidly and accurately detect SARS-CoV-2 nucleic acids in respiratory specimens. Sherlock Biosciences developed the SHERLOCK method (**s**pecific **h**igh-sensitivity **e**nzymatic **r**eporter un**lock**ing), which utilizes the Cas13a enzyme (Kellner et al., 2019). In contrast, Mammoth Biosciences developed the DETECTR method (**D**NA **e**ndonuclease **t**arg**e**ted **C**RISPR ***t****rans* **r**eporter), which utilizes the Cas12a enzyme (Chen et al., 2018). Cas13a and Cas12a have a “collateral cleavage” activity triggered by a target-dependent binding between the Cas-guide RNA complex (CRISPR complex) and the targeted sequence. This event activates the nuclease enzyme activity of the Cas, followed by the cleavage of the nucleic acid reporter and the generation of a detectable signal. SHERLOCK-based detection method recently received FDA EUA. It combines Cepheid automated RT-PCR for SARS-CoV-2 nucleic acid amplification followed by collateral cleavage activity of the CRISPR complex programmed to target the SARS-CoV-2 sequences; the resultant fluorescent signal is then detected by a plate reader. The whole process takes less than an hour without elaborate instrumentation. It can detect targeted sequences at a concentration as low as 6.75 copies/μL; it also has 100% specificity and sensitivity with the absence of cross-reactivity with sequences of high homology (Zhang et al., 2020a). On the other hand, DETECTR-based detection uses an additional reporter dye (Fluorescein amidite, FAM), which produces color when cut. DETECTR combines RT-LAMP for SARS-CoV-2 targeted sequences amplification followed by lateral flow assay for visual read-out detection. The assay time is 30–40 min and has 95% sensitivity and 100% specificity (Broughton et al., 2020). According to the initial validation testing of the cutting-edge CRISPR-based detection platforms, these tests have great potential for diagnosis. They do not require heavy instrumentation, and the results can be read quickly by a paper strip or a plate reader with minimal cost and high sensitivity and specificity.

## Serology-Based Immunoassays (Antibodies Detection)

**Figure 4:**
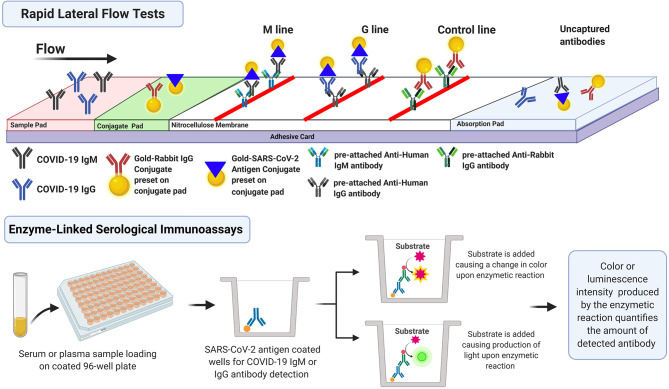
Serology-based immunoassays for detection of anti-SARS-CoV-2 antibodies.

Apart from molecular diagnostics and nucleic acid detection, various assays have been developed by several companies to test for antibodies produced in response to SARS-CoV-2 infection. Antibody-based tests are relatively cheap, easy to operate, and require less technical expertise compared to molecular-based assays. The dynamics of antibodies production may provide a larger window for detecting and monitoring current and past SARS-CoV-2 infections. Current serology-based tests include rapid lateral flow immunoassay tests also known as immunochromatographic assays (ICA), enzyme-linked immunosorbent assay (ELISA), automated chemiluminescence microparticle immunoassay (CMIA) among others. This section is a summary of two main approaches used to detected immunoglobulins produced against SARS-CoV-2 that received FDA EUA and/or CE Marking. These antibody detection tests are summarized in Supplementary Table 1, and the workflow is illustrated in Figure 4.

### Serology-Based Rapid Lateral Flow Immunoassay for Antibody Detection

Lateral flow assay allows for the qualitative detection (positive or negative) of antibodies found in a blood sample. This test is easy to perform, inexpensive to develop, and provides results within minutes. This gives lateral flow assays great advantage for rapid diagnostic testing of COVID-19 by testing for the presence of different isotypes of antibodies that target different SARS-CoV-2 proteins (N and S proteins among others). Several assays, referred to as total antibody assays, employed the detection of all isotypes (IgM, IgG, and IgA), while others targeted IgM and IgG exclusively. Both approaches were investigated by Lou et al. (2020) who compare the performance of 3 different lateral flow assays targeting IgG, IgM, and total antibody. They reported that the first detectable serological markers were total antibody, followed by IgM then IgG (9, 10, and 12 days after symptom onset). They also noted that the total antibody assay had the highest sensitivity among the others (98%) 2 weeks post-symptom onset.

The lateral flow assay requires a minimal sample volume of around 20 μL of blood or 10 μL of serum/plasma. It also does not require bulky instrumentation or trained personnel (Vashist, 2020). In this assay, the sample is placed on the absorbent sample pad of the lateral flow test strip and is allowed to migrate through the conjugate release pad which contains the SARS-CoV-2 antigen conjugated to colored or fluorescent particles, most commonly colloidal gold. The antigen is usually the receptor-binding domain of SARS-CoV-2 spike protein or nucleocapsid protein. If the patient was infected with SARS-CoV-2 and had the specific antibodies, then the sample now containing IgG and/or IgM antibodies bound to the conjugated antigens migrates to the detection zone. This zone contains anti-human IgG and IgM lines (test lines) that capture the antigen-antibody complexes, in addition to a control line. The read-out, which appears as lines with varying intensities can be assessed using a dedicated reader or by eye (Koczula and Gallotta, 2016).

Several lateral flow assays for COVID-19 IgG and IgM antibodies have been FDA EUA approved, including qSARS-CoV-2 IgG/IgM Rapid Test developed by Cellex which targets the S and N proteins and gives qualitative results within 15–20 min with 93.8% positive percent agreement (PPA) and 96% negative percent agreement (NPA) (FDA, 2020c; Mathur and Mathur, 2020). AutoBio Diagnostics also developed anti-SARS-CoV-2 rapid test that tests for IgG only and targets the spike protein with reported 88.15% PPA and 99.04% NPA with previously PCR tested samples (Mathur and Mathur, 2020).

### Enzyme Linked Serology-Based Immunoassays for Antibody Detection

COVID-19 enzyme-linked serology-based immunoassays are rapidly emerging as tools for surveying exposed individuals, including those who are asymptomatic or have recovered. These tests are at increased demand to better quantify the number of COVID-19 cases for epidemiological and surveillance purposes. Enzyme-linked immunoassays are usually performed in a laboratory setting and can be either qualitative or quantitative; specimens can be plasma or serum specimens. ELISA is the conventional platform for assessing SARS-CoV-2 exposure; it can detect antibodies at the lowest concentrations (~picogram/mL ranges). It is a microwell, plate-based assay used for the detection and quantification of antibodies produced against the viral infection. The well plate is coated with a deactivated virus or a recombinant viral antigen, typically, the S protein, (RBD) protein, or the N protein. Patient samples are then added to the well and the plate is incubated. If the patient has antibodies against the viral protein, an antibody-antigen complex is formed, and the complex can be detected by an enzyme-linked or a fluorescently tagged secondary antibody designed against the human antibodies (anti-human IgG/IgA/IgM). A colorimetric or a fluorescent signal is produced according to the type of secondary antibody used; the intensity of the signal reflects the quantity of the antibodies present within the sample after adjusting for the dilution factor. The whole process typically takes 2–5 h, but up to 96 samples can be tested at a time (Wild, 2013). It is worth noting that ELISA cannot tell if the detected antibodies are active or effective against the viral infection. Such information can be depicted by plaque reduction neutralization tests that involve cell culture, viral infection, and viral replication assessment. Several ELISA kits have been FDA approved for emergency use in COVID-19 diagnosis. For instance, Bio-Rad Laboratories Inc. designed the Platelia SARS-CoV-2 total Ab Assay which detects IgG, IgA, and IgM; it has 92% PPA and 99.5% NPA with previously PCR tested samples.

Another serology-based enzyme-linked platform is the CMIA (chemiluminescent microparticle immunoassay) that has low limits of antibody detection (~zeptomole 10^−12^ mol) and involves chemiluminescence for signal read-out; it is a modified advanced form of ELISA. CMIA offers several advantages over ELISA in terms of cost, sensitivity, LOD, and reproducibility. Also, the testing time is reduced due to shorter incubation periods and reaction times, where both processes are fully automated by digital analyzers (Qin and Jin-Ming, 2015). On the contrary, similar to ELISA, the test is typically qualitative and quantitative; it is also laboratory-based, and the samples are either plasma or serum specimens. The test relies on magnetic protein-coated microparticles used as a solid support to coat the wells with the viral antigen of interest. The full assay takes 1–2 h to perform with incubation steps similar to the conventional ELISA. The substrate is added to the enzyme-linked secondary antibody, which is bound to the antibody-antigen complex, and produces a light (radiance) signal. The luminescence is used to quantify the amount of antibodies present in the sample. The use of streptavidin-coated magnetic beads with several biotinylated viral antigens allows high sensitivity multiplexing if several antibodies are needed to be detected at the same time within the same sample (Cinquanta et al., 2017). A very promising CMIA-based FDA approved assay is the SARS-CoV-2 IgG Assay by Abbott Laboratories Inc. It has more than 96% PPA and 99.63% NPA in samples taken 14 days post-symptoms onset. On the other hand, chemiluminescence-based assays can be coupled with electrical voltage instead of enzyme-linked secondary antibodies for signal emission. Roche Diagnostics is a pioneer in this field with its Elecsys Anti-SARS-CoV-2 kit. Elecsys is an electrochemiluminescence microparticle immunoassay (ECMIA) that uses the Cobas immunoassay analyzer and takes only 18 min for the testing time. It uses the double antigen sandwich format to detect IgM, IgG, or IgA antibodies with high affinity. The first recombinant antigen is biotinylated while the second is coupled with ruthenium complex. When the double antigen-antibody sandwich complex is attained, streptavidin-coated microparticles are added to bind to the biotinylated antigen. The mixture is then aspirated to the measuring cell and microparticles are magnetically captured on the surface of the electrode. Application of voltage to the electrode induces radiance emission from the ruthenium and the light is measured by a photomultiplier. Elecsys Anti-SARS-CoV-2 has a high accuracy of targeting high affinity antibodies with 99.81% specificity and 100% sensitivity from samples taken 14 days post-symptoms onset; in addition, it is capable of running up to 300 serology tests/h depending on the type of the analyzer.

Currently, over 40 manufacturers have developed serology-based tests and received the FDA EUA. The FDA requires the laboratories to validate their assay as deemed appropriate (FDA, 2020a); thus, the absence of FDA oversight on these tests is a concern if we take into consideration the high variability of the proposed test formats (ICA, ELISA, CLIA, ECMIA) and the differences in the antibody class(es) detected. As such, along with the novelty of SARS-CoV-2 and our limited information regarding the human immune response to it, it is worth noting that serology-based immunoassays have not been fully evaluated and their true clinical performance is mostly unknown. In addition, according to the manufacturer's data sheets, the performance of serology immunoassays is frequently evaluated in comparison to results obtained by the RT-PCR molecular diagnostic assay; given the differences in both assays format along with the design and targets, serology negative results do not rule out the possibility of infection. Should mass testing and screening be recommended, it is vital for laboratories considering serology-based immunoassays to perform thorough verification studies to ensure the appropriate clinical performance of these tests and the accuracy of the obtained results.

## Antigen-Based Assays for SARS-CoV-2 Detection

Antigen-based diagnostics detect protein fragments on or within the virus rather than viral nucleic acids in specimens collected from NP swabs or nasal cavity. This type of testing is a rapid point-of-care platform that can detect active infections within 15 min compared to hours with RT-PCR diagnostics. These tests can be mass-produced at low cost and have a simpler setup compared to RT-PCR tests (Chen et al., 2015). Although antigen-based tests are very specific, they are not as sensitive as RT-PCR tests (CDC, 2020). This is attributed to the fact that rapid antigen testing sensitivity correlates directly with the viral load that is maximal during the first week of onset only. Thus, antigen-based testing has a relatively small window of detection during the incubation period of infected individuals. RT-PCR is more sensitive because its LoD is as low as 10^2^ genomic copies/mL, while that of rapid antigen tests is ~10^5^ genomic copies/mL when correlating the amount of viral proteins to viral nucleic acid quantity (Nash et al., 2020; Vogels et al., 2020b).

So far, EUA was issued to four antigen-based detection diagnostic tests: Sophia 2 SARS Antigen FIA (Quidel Corporation, USA), BD Veritor System for Rapid Detection of SARS-CoV-2 (Becton, Dickinson and Company (BD), USA), LumiraDx SARS-CoV-2 Ag Test (LumiraDx UK Ltd., UK) and BinaxNOW COVID-19 Ag Card (Abbott Diagnostics Scarborough, Inc.). These antigen-based detection tests are qualitative lateral flow immunoassays used to detect viral proteins, the N protein, in upper respiratory specimens during the acute phase of infection. A respiratory specimen is obtained (usually a NP or a nasal swab) and suspended in an extraction solution to liberate the viral antigens. The sample is then dispensed into the test cassette well where it migrates by capillary action to regions containing antibodies against the viral antigen. If SARS-CoV-2 antigens are present, a signal is produced and is detected. The signal can be colorimetric and read visually as in BD Veritor System for Rapid Detection of SARS-CoV-2 and BinaxNOW COVID-19 Ag Card tests, or it can be fluorescent (for higher sensitivity) and read by digital analyzers as in Sophia 2 SARS Antigen FIA and LumiraDx SARS-CoV-2 Ag Test. These tests are automated and are authorized for use in high and moderate complexity laboratories certified by CLIA, as well as for point-of-care testing by facilities operating under a CLIA Certificate of Waiver.

Among the tens of different tools developed and proposed for COVID-19 detection, by the time this review has been written, only tests belonging to five approaches received the FDA, and all are presented and discussed in this review paper. These approaches differ on various levels starting with the technology itself, specimen collection and targets, as well as sample processing. These differences are presented and compared in Table 1.

**Table 1:** Comparisons of different COVID-19 diagnostic assays.

	**RT-PCR assay**	**Isothermal amplification assay**	**CRISPR-based assay**	**Lateral flow assay**	**Enzyme linked tests**
Target	ORF1ab, RdRp, E, S, and N genes	ORF1ab, RdRp, and N genes	ORF1ab, E, and N gene (Broughton et al., 2020)	-IgG and IgM antibodies -N protein (antigen)	IgG, IgM, and IgA antibodies
Specimen	Upper and lower respiratory specimens, saliva, bronchoalveolar lavage fluids	Upper and lower respiratory specimens, nasal, and throat swabs	Nasopharyngeal or oropharyngeal swabs and bronchoalveolar lavage fluids	-Serum, Saliva or Nasal swab fluids (antibodies) -Nasopharyngeal or oropharyngeal swabs (antigen)	Plasma or serum samples
Day post-symptoms where the target is max	First week after symptom onset (Tan et al., 2020)	First week after symptom onset (Tan et al., 2020)	First week after symptom onset (Tan et al., 2020)	-IgM: 10–35 days after onset -IgG: 10 days after onset (Ma et al., 2020) -Antigen: before or at symptoms onset	-IgM: 10–35 days after onset -IgG: 10 days after onset (Ma et al., 2020)
Sample preparation	-RNA extraction -Target gene amplification -Florescent signal readout of the amplification signals	-15-min RNA sample preparation in sample buffer -Target gene isothermal amplification -Florescent readout signal of the amplification signals	-RNA extraction -Target gene isothermal amplification -Target recognition and cleavage -Lateral flow visual readout or fluorescent readout by a plate reader	Plasma or serum separation from specimen by centrifugation and transfer to the test cassette for lateral flow capillary movement (antibodies) Extraction of viral antigen from swabs by special media and loading to test cassette for later flow capillary movement (antigen) Lateral flow visual readout or fluorescent readout	-Plasma or serum separation from specimen by centrifugation or sampling tubes with separation gel -sample processing and coating with magnetic particles -Signal readout by a plate reader or a photomultiplier
Control Sample	Human RNase P gene (internal control)	N.A.	RNase P gene (positive control) (Broughton et al., 2020)	C line positive control band	N.A.
Number of samples per run	Device dependent (1 sample per run—tens-hundreds of samples per run)	1 sample/13 min	N.A.	1 sample per run	100–170 results/h
Assay-to-result time	120 min (excluding RNA extraction)	15–60 min 5–13 min	30–40 min (DETECTR) ~60 min (SHERLOCK)	15–20 min	35 min
Sample-to-result time	8 h	h	45 min−1 h	20–30 min	1–5 h
Result type	Quantitative	Qualitative	Qualitative	Qualitative	Quantitative
Sensitivity	94.34–100%	100%	95%	82–93.8% (antibodies) 80% (antigen)	87.5–100%
Specificity	94.87–100%	100%	100%	90.63–100% (antibodies) 100% (antigen)	95–100%
Instrumentation	High	Moderate	Low	Low	Low
Automation	Possible automation	Automated	Automated	Possible automation	-ELISA: semiautomated -CLIA: Automated
Point-of-care	Possible	Yes	Yes	Yes	No
Scale of production (easy, hard)	Hard	Hard	Easy	Easy	Easy
Cost (high, low)	High	Moderate-low (Cantera et al., 2019)	Low	Low	High-moderate

## Testing Sensitivity vs. Rapidity

The current reliance on high accuracy detection of COVID-19 cases for community surveillance and safe reopening of societies has put testing sensitivity and rapidness under the spotlight. The gold standard for COVID-19 detection is the molecular RT-PCR which has low LoD and high sensitivity, but at the same time, it is expensive, laboratory-based and often takes at least 24–48 h for sample-to-answer-reporting time.

New developments in COVID-19 diagnostic technology are focused on reducing test cost and sample-to-result time to expand testing frequency and lessen testing turnaround time from days to minutes. However, the sensitivity of these tests is not comparable to that of RT-PCR making it almost impossible to get the FDA EUA/CE marking approval. However, such restrictions should be reconsidered when taking into account both viral kinetics and RT-PCR sensitivity. As discussed earlier, patients undergo a period of peak in viral load and infectiousness followed by a rapid decline in viral levels and clearance. Given RT-PCR amplification is exponential, it can detect viral RNA at extremely low levels yielding high Ct values. Nevertheless, detection of low RNA levels may not hold much value as Singanayagam et al. (2020) showed. They demonstrated that the odds of recovering an infectious virus from patients decreased by 0.67 for each unit increase in Ct value. Additionally, only 8% of the samples with Ct > 35 yielded culturable virus (Singanayagam et al., 2020). These data were consistent with other studies by Bullard et al. (2020) and La Scola et al. (2020). Accordingly, a cheap, rapid, and robust test with acceptable sensitivity would be able to detect the viral infection at least at the contagious period of illness when the viral load is the highest and would help in halting and controlling the coronavirus pandemic.

Current attempts to improving testing accessibility and scalability in the Yale-NBA study [**S**urveillance **with I**mproved **S**creening and **H**ealth (SWISH) study] have led to the development of the SalivaDirect test. SalivaDirect uses a conventional quantitative nucleic acid-based detection technique (RT-PCR or RT-LAMP) but replaces the nucleic acid extraction step with a simple proteinase K and heat treatment step. Bypassing the conventional intricate RNA extraction method minimizes sample processing time and reagents usage. Thus, it lowers the sample processing cost and alleviates the testing demands. The SalivaDirect kit recently received the FDA EUA approval and has shown more than 94% sensitivity agreement with the CDC RT-qPCR assay as claimed in their preprint (Vogels et al., 2020a). Nonetheless, using saliva as a specimen and enzymes for RNA extraction does not make this test a “rapid” one because of the amplification and detection steps required.

To meet the needs of COVID-19 mass testing, a true rapid test must be done outside laboratory settings with minimal machinery and technical expertise is needed. Also, given these tests are required around the world with different geographies and related economical constraints, it is vital for these rapid diagnostic tests to be affordable and cheap so that people can use them more frequently. In addition, these tests must possess acceptable sensitivity to detect SARS-CoV-2 infection in its contagious stage to stem the spread of the virus via individual screening. These challenges are clear when examining RT-PCR's sensitivity compared to its rapidity. By the time the RT-PCR results are reported, infected patients may no longer be in their contagious state while they were in contact with other individuals in their contagious period. Thus, RT-PCR is not suitable for daily and weekly surveillance due to its high testing turnaround time. In fact, a team has modeled surveillance effectiveness, taking into consideration testing sensitivity, testing frequency, and sample-to-answer reporting time. Their model, published in a preprint, indicates that controlling the COVID-19 outbreak depends largely on the frequency and rapidness of testing, and is marginally enhanced by high testing sensitivity (Larremore et al., 2020).

In response to the COVID-19 pandemic and the shortage of laboratory-based testing capacity and reagents, various rapid and sensitive tests are currently in development, many of which are the antigen-based diagnostics. The viral proteins are detected in the sample if present in sufficient concentrations without the need for amplification. The currently FDA EUA approved antigen-based tests detect the presence of the N protein in the sample, thus the virus must be permeated first for antigen extraction. In addition, some tests use fluorescent signal readout for improved sensitivity, making them more expensive and dependent on digital analyzers, thus they cannot be performed outside laboratory settings. Yet, a better rapid antigen-based diagnosis is a test that detects the S protein directly, without the need for antigen extraction, in a lateral flow immunoassay platform with a colorimetric readout. E25Bio Company has developed such a test and submitted its work for the FDA EAU approval, but has not received it yet. If we accept rapid tests with moderate sensitivity that can detect viral antigens in their peak (active phase of infection), then we might be able to manage the coronavirus transmission and halt its pandemic.

## Discussion: Unmet Needs and Other Considerations of COVID-19 Diagnostic Platforms

The large deployment of various SARS-CoV-2 diagnostics in several countries has aided in curbing the spread and transmission of the COVID-19 pandemic. In fact, mass daily testing is required to slowdown the crisis by identifying active cases. Hence, this will aid in isolating active cases and their contacts, reducing the pressure on intensive care units and allowing containment of possible new outbreaks at the earliest of time. Despite the relatively large number of SARS-CoV-2 diagnostic tests and the remarkable speed of evolving new ones, the performance of currently available tools is still unclear and requires further verification and confirmation. The utility of these diagnostic testing is contingent on several factors mainly the type of testing, the resources required, the cost of the test, the sensitivity and specificity as well as the time needed to obtain results.

RT-PCR is the primary molecular armamentarium for the etiological diagnosis of COVID-19. This nucleic acid-based approach is inherently quantitative, but current SARS-CoV-2 tests are being promoted by manufacturers as qualitative (either positive or negative). The obtained results are only indicative of whether the virus or its genetic material is present at the time of the testing, but do not rule out the probability of past infection. Positive results indicate the presence of the viral genome but do not infer the infection status of the patient. The latter should be determined by clinical testing combined with clinical history and complementary diagnostic tools, such as cell culture or antigen-based tests.

An ideal genetic target would include at least one conserved region and one SARS-CoV-2 specific region. To date, the choice of the genetic sequence in the various RT-PCRs kits has not offered a unique advantage to diagnostics, but detecting only one viral gene instead of two, when two sequences are tested, has been conflicting. In addition, negative results in suspects do not rule out the possibility of infection. In fact, several factors could lead to false negatives, such as poor sample quality, improper sample handling and storage, or inappropriate sample collection timing (too early or too late) (To et al., 2020b; Williams et al., 2020). It is important to point out that viral shedding is related to the stage of illness and severity (Becherer et al., 2020). Besides, the lack of a standard sample control, a standard reference test, and the use of different sample collection and preparation protocols with the different LoD have hampered our understanding of the virus dynamics and the accuracy of the newly introduced molecular-based detection assays. These various molecular-based detection assays have been yielding different positive or negative agreement results when used in comparison to various RT-PCR due to the lack of a standard confirmatory RT-PCR kit for direct COVID-19 detection (Tahamtan and Ardebili, 2020). These differences are related to assay performance influenced by the specimen itself, the reagents and the primers used. In fact, several studies have documented that SARS-CoV-2 has been showing genetic diversity and evolution which might affect the RT-PCR results (Phan, 2020; Shen et al., 2020).

Similar to sample collection location and timing, sample storage, sample handling and sample viral inactivation tend to influence the diagnostic results and may yield false negatives in many cases. The collected clinical specimen should be stored promptly at 2–8°C, then processed immediately when shipped for RNA extraction to avoid RNA degradation. In addition, the specimen, if not used immediately, should be frozen in the transport media at −20 or ideally −70°C if a delay in sample processing is expected. Moreover, heat treatment of samples before RNA extraction at temperatures above 56°C for 30 min is not recommended; this may lead to sample inactivation and loss of detectable viruses in a sample. The latter would give false negatives especially in a weakly positive sample (Chen et al., 2020).

Given the aforementioned limitations, various integrated automated point-of-care molecular diagnostics are currently under development with some receiving EUA and aim to deliver rapid accurate results with low processing complexity. The enclosed system combining RNA extraction, amplification and detection within a sealed cartridge is suitable for testing without the need of biosafety cabinets and can be used to scale up testing with high throughput, such systems include ID Now by Abbott and Xpert Xpress SARS-CoV-2 test by Cepheid. In contrast to RT-PCR, some point-of-care tests do not use a positive human gene control. Thus, they can't determine whether a given sample contains sufficient viral RNA and may yield false negatives. In addition, isothermal amplification-based detection technology has low throughput but does not require sample transport, RNA extraction, or batching with other samples.

Complementary to molecular assays are immunoassays for COVID-19 epidemiological surveillance. They are good alternatives, especially in community clinics and small hospitals that do not have access to molecular diagnostics equipment and expertise. Immunoassays provide a cheap and rapid indirect measure of infection, confirming positive cases obtained by molecular testing. The detected IgM and IgG antibodies in clinical specimens are likely limited around the time of symptoms onset when the viral shedding is the highest and transmission rate is the maximal. The antibody responses to infection take several days or weeks to be detected, therefore negative results do not exclude infection especially if the person has been recently exposed to the virus. The issue lies with patients having low viral load and poor immune response; therefore, such assays might miss capturing the antibody or the antigen in the sample and provide false negative results. On the contrary, immunoassays cross-reactivity with other coronaviruses proteins or antibodies has been very common and yielded false-positive results in many cases (CDC, 2020). The assessment of immunoassays has been done in comparison to the gold standard RT-PCR and therefore understanding the performance and the sensitivity of such tests has not been straightforward. Instead, the comparison must include the evaluation of antibody profiles at the different infection stages (early infection vs. late infection vs. convalescent period) and among asymptomatic cases.

Although WHO recommended immunoassays when molecular diagnostic testing is not available, these tests may be useful for determining the immune status of exposed individuals and the overall spread of COVID-19. However, immunoassays are unlikely to be useful for screening or early diagnosis. As such, these diagnostic tests can be essential tools for risk management and public health. Quantitative immunological tests are also critical for identifying recovered individuals with enough antibody titers who could donate their convalescent plasma to current COVID-19 patients. All in all, rapid low-cost point-of-care diagnostics are currently in high demand to rapidly discover active cases, limit viral transmission between the individuals and predict epidemiological outcomes for disease surveillance.

## Conclusion

Lessons learned from the challenges presented by the COVID-19 testing will remain relevant for potential future management and mitigation of new viral outbreaks. This is especially true as new and innovative screening technologies were developed and deployed in record time. It has become evident that high accuracy, scalability, and rapid testing is essential in the fight against outbreaks, especially respiratory viral diseases that can be transmitted easily from person to person. Also, understanding the viral load over the course of the infection can have an impact on reducing false negatives. The same can be said for serological biomarkers. In addition, the deployment and frequency of testing across the globe can be influenced by the geographical and economic constraints in each country given the complicated workflow and price of some testing technologies, such as that of RT-PCR. Finally, managing the data for contact tracing in every country as few of these systems are connected to a global data base can very challenging. Shared data can aid in understanding level of infections within populations locally and across the world enabling improved resource management and better understanding of the rate of transmission.

## Author Contributions

MK, ZH, and SS developed the review outline, drafted and wrote the manuscript, and developed the figures. MK conceived the idea and supervised the content and writing. HZ critically contributed to the content and reviewed the manuscript to ensure accuracy and completeness. All authors contributed to the article and approved the submitted version.

## Conflict of Interest

The authors declare that the research was conducted in the absence of any commercial or financial relationships that could be construed as a potential conflict of interest.
