# Supplementary material for: COVID-19 in-vitro Diagnostics: State-of-the-Art and Challenges for Rapid, Scalable, and High-Accuracy Screening
Source: Front Bioeng Biotechnol. 2021 Jan 28;8:605702. doi: 10.3389/fbioe.2020.605702 (PMC7902018; doi:10.3389/fbioe.2020.605702)
Supplement: Supplementary file 1 [file Table_1.pdf]

In-Vitro Diagnostics for Rapid and High-Accuracy COVID-19 Screening.

Table 1. Diagnostic tests that received FDA EUA for emergency use in Clinical Laboratory Improvement Amendments (CLIA)-certified molecular diagnostics laboratories as of July 2020. **Approved (H):** FDA EUA, restricted to CLIA-certified molecular diagnostics laboratories to perform high complexity tests. **Approved (H, M):** FDA EUA, restricted to CLIA-certified molecular diagnostics laboratories to perform high and moderate complexity tests. **NP:** Nasopharyngeal, **OP:** Oropharyngeal, **AN:** Anterior nasal, **MT:** Mid-turbinate nasal, **BAL:** Bronchoalveolar lavage, **TA:** Tracheal Aspirate, **GC**: Genomic copies, **GE:** Genome equivalents, **PFU:** Plaque forming unit, **$TCID_{50}$:** Tissue culture infectious dose, **PPA:** Positive Percent Agreement (Sensitivity), **NPA:** Negative Percent Agreement (Specificity). PPA and NPA were extracted from the Instructions for Use (IFU) manual of each diagnostic tool (FDA, 2020b). EUA status was updated using FINDdx (FINDdx, 2020).

Manufacturer	Test name	Sample	Description	Biomarkers	LOD/Sample Volume	PPA and NPA	EUA Status
**RT-PCR for SARS-CoV-2 Detection**							
Sansure BioTech Inc.	Novel Coronavirus (2019-nCoV) Nucleic Acid Diagnostic Kit (PCR-Fluorescence Probing)	-NP, OP, AN and MT swabs	-RT-PCR (PCR-Florescence Probing) for qualitative detection of the SARS-CoV-2 RNA	ORF1ab and N genes	200 copies/mL	-PPA is 94.34% in 53 RT-PCR positive samples<br>-NPA is 98.96 % in193 RT-PCR negative samples	- Approved (H)<br>- CE Marked<br>- Australian Therapeutic Goods Administration authorization<br>- Singapore Health Sciences Authority
Bio-Rad Laboratories, Inc	Bio-Rad SARS-CoV-2 ddPCR Test	-NP, AN and MT swabs<br>sal and NP<br>h/aspirate	-Partition-based endpoint RT-PCR (RT-ddPCR: droplet digital PCR) test intended for the qualitative detection of SARS-CoV-2 RNA	N gene (N1 and N2 regions)	625 copies/mL	-PPA: 94.87%<br>-NPA: 94.87%	-Approved (H)<br>-CE marked approval<br>-RUO system and lab equipment
BioFire Diagnostics, LLC	BioFire Respiratory Panel 2.1 (RP2.1)	NP swabs	-Multiplexed PCR intended for the simultaneous qualitative detection and differentiation of SARS-CoV-2 RNA from multiple viral and bacterial respiratory organisms<br>-results in ~ 45 min	S and M genes	300 copies/mL	-PPA: 98%<br>-NPA: 100%	Approved (H, M)
OPTI Medical Systems, Inc.	OPTI SARS-CoV-2 RT PCR Test	-Nasal, NP and OP swabs<br>-Sputum, TA, BAL, nasal and NP aspirate/wash	-RT-PCR test for the qualitative detection of SARS-CoV-2 RNA	N gene (N1 and N2 regions)	- 0.7 copies/µL in sputum samples<br>- 0.9 copies/µL in NP swab samples	100%	- Approved (H)<br>- CE marked
BioMérieux SA	SARS-COV-2 R-GENE®	-NP, OP, AN and MT swabs<br>-Nasal aspirate/wash<br>-BAL	-RT-PCR test for the qualitative detection of SARS-CoV-2 RNA	RdRp, E and N genes	380 GC/mL	100%	Approved (H)
Fast Track Diagnostics Luxembourg S.á.r.l. (a Siemens Healthineers Company)	FTD SARS-CoV-2	-Nasal, NP, OP swabs<br>- Nasal and NP wash/aspirate<br>-BAL	-RT-PCR test for the qualitative detection of SARS-CoV-2 RNA	ORF1ab and N genes	0.0023 $TCID_{50}$/mL	100%	- Approved (H)<br>- CE marked
LabGenomics Co., Ltd.	LabGun^{TM} COVID-19 RT-PCR Kit	-NP, OP, AN and MT swabs	-RT-PCR test for the qualitative detection of SARS-CoV-2 RNA	RdRp and E gene	20 GC/µL	100%	-Approved (H)<br>-CE marked

		-Nasal and NP wash/aspirate<br>-Sputum					- Australian Therapeutics Administration authorization
Rheonix, Inc.	Rheonix COVID-19 ™ MDx Assay	-NP, OP, AN, MT and nasal swabs<br>-Nasal wash/aspirate BAL	-RT-PCR test for the qualitative detection of SARS-CoV-2 RNA	N gene (N1 region)	625 GE/mL	100%	Approved (H)
SEASUN BIOMATERIALS	U-TOP™ COVID-19 Detection Kit	-OP, NP, AN, MT and nasal swabs<br>-Nasal and NP wash/aspirate<br>-Sputum	-RT-PCR for qualitative detection of the SARS-CoV-2 RNA with 95% positivity rate	Two regions ORF1ab and one region N gene	1 copy/µL		- Approved (H)<br>- CE Marked<br>- Singapore Health Sciences Authority
SD Biosensor, Inc.	STANDARD M nCoV Real-Time Detection kit	-NP, OP, nasal, MT and nasal swabs<br>-Sputum	-RT-PCR test for the qualitative detection of SARS-CoV-2 RNA<br>-Results in ~90 min	RdRp and E genes	- 0.25 copies/µL for upper respiratory specimens and 0.125 copies/µL for lower respiratory specimens on the CFX95 amplification system<br>-0.5 copies/µL for upper respiratory specimens and 0.25 copies/µL for lower respiratory specimens on the LC480 amplification system	-PPA is 100% in 30 samples<br>-NPA is 100% in 30 samples	- Approved (H)<br>- CE Marked<br>- Brazilian Health Regulatory Agency authorization<br>-Philippines FDA
Altona Diagnostics GmbH	RealStar® SARS-CoV-2 RT-PCR Kit U.S	-NP, OP, AN, MT and nasal swabs<br>-Nasal wash/aspirate	-RT-PCR test for the qualitative detection of SARS-CoV-2 RNA	E and S genes	- 625 copies/mL<br>-10^{-1} PFU/mL	-PPA is 97.8%<br>-NPA is 97.3%	- Approved (H)<br>- CE marked
Seegene, Inc.	Allplex™ 2019-nCoV Assay	-NP, OP, AN and MT swabs<br>-Sputum	-RT-PCR test for the qualitative detection of SARS-CoV-2 RNA<br>-results in ~2 hours excluding RNA extraction	RdRp, N gene and E genes	-1250 copies/mL is using CFX96TM amplification system<br>-4167 copies/mL is using CFX96 TouchTM amplification system	- PPA is 100% in NP, OP and sputum samples<br>-NPA is 93.07% For NP and OP samples, and 100% in sputum samples	- Approved (H)<br>- CE Marked<br>- Australian Therapeutic Goods Administration authorization<br>- Singapore Health Sciences Authority<br>- Health Canada authorization<br>- Brazilian Health Regulatory Agency authorization
Trax Management Services Inc.	PhoenixDx® 2019-CoV	-Nasal, NP and OP swabs<br>-BAL	-RT-PCR test for the qualitative detection of SARS-CoV-2 RNA	RdRp and E genes	100 copies/mL	100%	Approved (H)
OSANG Healthcare	GeneFinder™ COVID-19 Plus RealAmp Kit	-NP, OP, nasal and MT swabs<br>-BAL<br>-Sputum	-RT-PCR test for the qualitative detection of SARS-CoV-2 RNA	ORF1ab, N and E genes	0.5 copies/µL	100% in NP, OP and BAL swabs	Approved (H)

In-Vitro Diagnostics for Rapid and High-Accuracy COVID-19 Screening.

Fosun Pharma USA Inc.	Fosun COVID-19 RT-PCR Detection Kit	AN, MT, NP and OP swabs<br>-sputum<br>-lower respiratory tract aspirates<br>-BAL<br>-Nasal and NP wash/aspirate	- RT-PCR test for the qualitative detection of SARS-CoV-2 RNA	ORF1ab, N and E genes	3000 copies /mL	-PPA: 99.51%<br>-NPA: 96.44%	Approved (H)
KorvaLabs Inc.	Curative-Korva SARS-Cov-2 Assay	-OP, NP and nasal swabs<br>-Oral fluid specimens	-RT-PCR test for the qualitative detection of SARS-CoV-2 RNA	N gene (N1 and N2 regions)	200 copies /mL	100% in NP swabs	Approved (H)
GenoSensor, LLC	GS™ COVID-19 RT-PCR KIT	NP, OP, nasal and MT swabs	-RT-PCR test for the qualitative detection of SARS-CoV-2 RNA with 95% positivity rate for lowest viral concentration	OFR1ab, E and N genes	1 copy/µL	100%	Approved (H)
Maccura Biotechnology (USA) LLC	SARS-CoV-2 Fluorescent PCR Kit	upper respiratory specimens (e.g., OP swabs, NP swabs, nasal swabs, and MT swabs)	-RT-PCR test for the qualitative detection of SARS-CoV-2 RNA with 95% positivity rate for lowest viral concentration	OFR1ab, E and N genes	1000 copies/mL	-PPA: 100%<br>-NPA: 96.7%	-Approved (H)<br>-CE marked
DiaCarta, Inc	QuantiVirus™ SARS-CoV-2 Test kit	-NP and OP swabs<br>-Sputum	-RT-PCR test for the qualitative detection of SARS-CoV-2 RNA with 95% positivity rate for lowest viral concentration	OFR1ab, E and N genes	100-200 copies/mL depending on the amplification platform used	-PPA: 95%<br>-NPA: 100%	Approved (H)
Becton, Dickinson and Company	BD SARS-CoV-2 Reagents for BD MAX™ System	Nasal, NP and OP swabs	- RT-PCR test for the qualitative detection of SARS-CoV-2 RNA	N gene (regions N1 and N2)	40 GE/mL	-PPA: 95%<br>-NPA: 100%	Approved (H, M)
InBios International, Inc	Smart Detect™ SARS-CoV-2 rRT-PCR Kit	NP, AN and MT swabs	- RT-PCR test for the qualitative detection of SARS-CoV-2 RNA	OFR1ab, E and N genes	10-12.5 GE/reaction depending on the used amplification platform	-PPA: 100%<br>-NPA: 96.7%	Approved (H)
Gnomegen LLC	Gnomegen COVID-19 RT-Digital PCR Detection Kit	Nasal, NP and OP swabs	- Digital RT-PCR test for the qualitative detection of SARS-CoV-2 RNA<br>-results in ~3 hours	N gene (regions N1 and N2)	8 copies/reaction	100%	Approved (H)
Co-Diagnostics, Inc.	Logix Smart Coronavirus Disease 2019 (COVID-19) Kit	-BAL, sputum and TA<br>- NP and OP swab	- RT-PCR test for the qualitative detection of SARS-CoV-2 RNA<br>-results are in 63-90 mins	RdRp gene	4.29 copies/µL	100%	-Approved (H)<br>-CE marked
ScienCell™ Research Laboratories	ScienCell SARS-CoV-2 Coronavirus Real-time RT-PCR (RTqPCR) Detection Kit	-Nasal, NP and OP swabs<br>-BAL	- RT-PCR test for the qualitative detection of SARS-CoV-2 RNA	N gene (viral) and RPP30 gene (human, internal control to assess specimen quality)	$10^{0.5}$ copies/µL	100%	Approved (H)
Luminex Corporation	ARIES SARS-CoV-2 Assay	NP swabs	-Multiplexed RT-PCR test for the qualitative detection of SARS-CoV-2 RNA	ORF1ab and N genes	7.5×10^4 GE/mL	100%	Approved (H, M)

			-can run up to 144 tests per day<br>-time-to-results is ~2 hours with minimal hands-on time for samples processing				
Becton, Dickinson and Company (BD)	BioGX SARS-CoV-2 Reagents for BD MAX System	NP and OP swabs	- RT-PCR test for the qualitative detection of SARS-CoV-2 RNA<br>-Fully automated system that can process up to 24 samples simultaneously<br>- Results in 2-3 hours	N gene (N1 and N2 regions)	40 GE/mL	100%	Approved (H, M)
Ipsum Diagnostics, LLC	COV-19 IDx assay	NP and OP swabs	- RT-PCR test for the qualitative detection of SARS-CoV-2 RNA	N gene	8.5 copies/µL	100%	Approved (H)
QIAGEN GmbH	QIAstat-Dx Respiratory SARS-CoV-2 Panel	NP swabs	-Multiplex RT-PCR for the qualitative detection and differentiation of SARS-CoV-2 RNA from other types and subtypes of viruses<br>-Results in ~1 hour	RdRp and E genes	500 copies/mL	100%	- Approved (H, M)<br>-CE marked<br>-Health Canada authorization<br>-Philippines FDA<br>- Australian Therapeutics Administration authorization
NeuMoDx Molecular, Inc.	NeuMoDx SARS-CoV-2 Assay	Nasal, NP and OP swabs	- RT-PCR test for the qualitative detection of SARS-CoV-2 RNA<br>-Results in ~ 1 hour	N and Nsp2 (non-structural protein 2) genes	150 copies/mL	100%	- Approved (H, M)<br>- CE marked<br>- Singapore Health Sciences Authority
Luminex Molecular Diagnostics, Inc.	NxTAG CoV Extended Panel Assay	NP swabs	- RT-PCR test for the qualitative detection of SARS-CoV-2 RNA<br>-highly scalable mass production and cost-effective<br>-Can process up to 96 samples in around 4 hours	ORF1ab, E and N genes	5000 GE/mL	100%	Approved (H)
BGI Genomics Co. Ltd	Real-Time Fluorescent RT-PCR Kit for Detecting SARS-2019-nCoV	-OP, NP, AN, MT and nasal swabs<br>-Nasal wash/aspirate<br>-BAL	- Fluorescent RT-PCR test for the qualitative detection of SARS-CoV-2 RNA<br>- Results in ~3 hours	ORF1ab gene	100 copies/mL	-PPA: 88.1%<br>-NPA: 99.6%	- Approved (H)<br>- CE marked
Avellino Lab USA, Inc.	AvellinoCoV2 test	NP and OP swabs	- RT-PCR test for the qualitative detection of SARS-CoV-2 RNA	N gene (N1 and N3 regions)	55 copies/µL	100%	Approved (H, M)
PerkinElmer, Inc.	PerkinElmer New Coronavirus Nucleic Acid Detection Kit	NP and OP swabs	- RT-PCR test for the qualitative detection of SARS-CoV-2 RNA	ORF1ab and N genes	-9.307 copies/mL for the ORF1ab target<br>-30.467 copies/mL for the N target. (LoD varies according to specimen origin and amplification system)	100%	-Approved (H)<br>-CEmarked
Mesa Biotech Inc.	Accula SARS-Cov-2 Test	Nasal swabs	- RT-PCR and lateral flow technology test for the qualitative detection of SARS-CoV-2 RNA	N gene	100 copies/reaction	100%	-Approved (H, M)<br>-Authorized for distribution and use in patient care settings under a

			-Reaction time is 30 minutes<br>-Palm-sized system that can be used in non-laboratory-based conditions				CLIA Certificate of Waiver (Certificate of Compliance)
BioFire Defense, LLC	BioFire® COVID-19 Test	NP swabs	-Multiplexed RT-PCR test for the qualitative detection of SARS-CoV-2 RNA	ORF1ab and ORF8 gene	$3.3x10^2$ GC/mL	-PPA: 80%<br>-NPA: 100%	- Approved (H, M)<br>- CE marked
Cepheid®	Xpert® Xpress SARS-CoV-2 test	-NP, OP, nasal, and MT swabs<br>-Nasal wash/ aspirate	-Automated rapid RT-PCR test for the qualitative detection of SARS-CoV-2 RNA<br>-Delivers results in 2 hours from sample collection to test results<br>- Assay reaction time is 30 minutes with less than 1 min of hands-on sample preparation	E and N (N2 region) genes	250 copies/reaction	100%	-Approved (H, M)<br>-Authorized for distribution and use in patient care settings under a CLIA Certificate of Waiver (Certificate of Compliance)<br>-CE marked<br>-Health Canada authorization<br>-Philippines FDA<br>- Brazilian Health Regulatory Agency authorization<br>- Australian Therapeutics Administration authorization
Primerdesign Ltd.	Primerdesign Ltd COVID-19 genesig® Real-Time PCR assay	-Nasal and OP swabs<br>-BAL	- RT-PCR test for the qualitative detection of SARS-CoV-2 RNA	ORF1ab gene	0.33 copies/µL	-PPA is 94.7% in 38 positive samples with 1-2xLOD of viral concentration and 100% in 12 samples with 3-5x LoD viral concertation<br>-NPA is 100% in 50 negative samples	Approved (H)
GenMark Diagnostics, Inc.	ePlex® SARS-CoV-2 Test	NP swab specimens (NPS) eluted in viral transport media (VTM) specimen	-Automated multiplexed qualitative nucleic acid detection of SARS-CoV-2<br>-Less than 2 minutes of sample preparation and less than 2 hours for results	N protein	10^5 copies/mL	-PPA: 94.4%<br>-NPA: 100%	Approved (H, M)
DiaSorin Molecular LLC	Simplexa COVID-19 Direct assay	-NP and nasal swabs<br>-BAL	- RT-PCR test for the qualitative detection of SARS-CoV-2 RNA<br>-Results within 1 hours excluding RNA extraction	ORF1ab and S genes	-500 copies/mL for NP samples<br>-242 copies/mL for Nasal swabs samples	100%	-Approved (H, M)<br>-CE marked
Abbott Molecular	Abbott RealTime SARS-CoV-2 assay	-NP and OP swabs<br>-Nasal swabs, self-collected at a health care location or collected by a healthcare worker	- RT-PCR test for the qualitative detection of SARS-CoV-2 RNA	RdRp and N genes	100 copies/mL	-PPA: 94.7%<br>-NPA: 100%	Approved (H)
Quest Diagnostics Infectious Disease, Inc.	Quest SARS-CoV-2 rRT-PCR	-NP and OP swabs<br>-Sputum	-Results are obtained in less than 2 days	N gene (N1 and N3 regions)	136 copies/mL	100%	Approved (H)

		-TA<br>-BAL					
Quidel Corporation	Lyra® SARS-CoV-2 Assay	NP and OP swabs	-multiplexed RT-PCR test for the qualitative detection of SARS-CoV-2 RNA<br>-results are obtained in 75 mins excluding RNA extraction	pp1ab (non-structural polyprotein) gene	0.8 GC/µL	100%	-Approved (H)<br>-CE marked<br>-Health Canada authorization
Laboratory Corporation of America (LabCorp)	COVID-19 RT-PCR Test	-Nasal, NP and OP swabs<br>-Sputum<br>-BAL<br>-Nasal and NP	- multiplexed RT-PCR test for the qualitative detection of SARS-CoV-2 RNA<br>-Coupled with Pixel by LabCorp™ COVID-19 test home collection kit to self-collect nasal swab specimens at home by individuals	N gene (N1, N2, and N3 regions)	6.25 copies/µL for NP swabs and 12.5 copies/µL for BAL swabs	100%	Approved (H)
Hologic®, Inc.	Panther Fusion SARS-CoV-2	NP, nasal, andOP swabs and lower respiratory tract specimens	- Multiplex RT-PCR test for the qualitative detection of SARS-CoV-2 RNA<br>-Results are obtained in less than 3 hours in each panther fusion system<br>-The system can process up to 1150 tests in 24 hours	ORF1ab, S and N genes	10 GE/reaction	100%	Approved (H)
Thermo Fisher Scientific, Inc.	TaqPath™ COVID-19 Combo Kit	-NP, OP, nasal and MT swabs<br>-NP aspirate<br>-BAL	- Multiplex RT-PCR test for the qualitative detection of SARS-CoV-2 RNA<br>-High throughput system: complete RNA extraction and RT-PCR of up to 94 samples under 3 hours 200	ORF1ab gene (regions 1 and 2)	10^{-2} $TCID_{50}$/mL	100%	- Approved (H)<br>- CE Marked<br>- Australian Therapeutic Goods Administration authorization<br>- Singapore Health Sciences Authority
Roche Molecular Systems, Inc. (RMS)	cobas® SARS-CoV-2	Nasal, NP and OP swabs	- RT-PCR test for the qualitative detection of SARS-CoV-2 RNA<br>- Automated sample preparation (RNA extraction and purification)<br>- Results in ~3.5 hours<br>-Can process up to 348 results using the cobas 6800 system or 1056 results using the 8800 system in 8 hours	ORF1ab and E genes	0.009 $TCID_{50}$/mL	100%	- Approved (H, M)<br>-CE marked
Wadsworth Center, New York State Department of Public Health's (CDC)	New York SARS-CoV-2 Real-time Reverse Transcriptase (RT)-PCR Diagnostic Panel	- NP and OP swabs<br>-Sputum	- RT-PCR test for the qualitative detection of SARS-CoV-2 RNA	N gene (N1 and N2 regions)	25 copies/reaction	100%	Approved (H)
Centers for Disease Control and Prevention's (CDC)	CDC 2019-nCoV Real-Time RT-PCR Diagnostic Panel (CDC)	-NP and OP swabs<br>-sputum<br>-lower respiratory tract aspirates	- RT-PCR test for the qualitative detection of SARS-CoV-2 RNA	N gene (N1 and N2 regions)	$10^{0.5}$-10^{0} copies/µL	100%	Approved (H)

In-Vitro Diagnostics for Rapid and High-Accuracy COVID-19 Screening.

		-BAL<br>-Nasal and NP wash/aspirate					
Abbott Molecular Inc.	Alinity m SARS-CoV-2 assay	-NP and OP swabs<br>-BAL	- RT-PCR test for the qualitative detection of SARS-CoV-2 RNA	RdRp and N genes	100 copies/mL	-PPA: 100%<br>-NPA: 96.5%	Approved (H, M)
1drop Inc.	1copy COVID-19 qPCR Multi Kit	-NP, OP, AN, MT and swabs<br>- Nasal and NP wash/aspirate	- RT-PCR test for the qualitative detection of SARS-CoV-2 RNA<br>- Results are obtained in less than 2 hours	E and RdRp genes	200 copies/mL	100%	Approved (H)
Applied DNA Sciences, Inc.	Linea COVID-19 Assay Kit	-Nasal, NP, OP, AN, MT and swabs<br>- Nasal and NP wash/aspirate	- RT-PCR test for the qualitative detection of SARS-CoV-2 RNA	S gene (S1 and S2)	1.25 copies/µL	-PPA: 98%<br>-NPA: 93%	Approved (H)
GeneMatrix, Inc.	NeoPlex COVID-19 Detection Kit	-NP, OP, MTand nasal swabs<br>-Sputum, TAs and BAL	-Multiplex RT-Real-time PCR Reagents for SARS-CoV-2 Detection	RdRp and N genes	50 copies/reaction for NP swabs and sputum	100%	-Approved (H)<br>-CE marked
Hologic, Inc.	Aptima® SARS-CoV-2 assay	NP, nasal, MT and OP swabs<br>- Nasal and NP wash/aspirate	- RT-PCR test for the qualitative detection of SARS-CoV-2 RNA	ORF1ab	0.01 $TCID_{50}$/mL	-PPA: 100%<br>-NPA: 98.2%	Approved (H)
Assurance Scientific Laboratories	Assurance SARS-CoV-2 Panel	-Nasal, NP and OP swabs	- RT-PCR test for the qualitative detection of SARS-CoV-2 RNA<br>-The test is coupled with a self-collection kit	N gene (N1 and N2 regions)	-29 copies/µL for N1 target<br>-9 copies/µL for N2 target	100%	Approved (H)
Fulgent Therapeutics, LLC	Fulgent COVID-19 by RT-PCR test	Nasal, NP and OP swabs	- RT-PCR test for the qualitative detection of SARS-CoV-2 RNA<br>-The test is coupled with a self-collection kit	N gene	5 copies/ µL	100%	Approved (H)
Quidel Corporation	Lyra® Direct SARS-CoV-2 Assay	Nasal, NP and OP swabs	- RT-PCR test for the qualitative detection of SARS-CoV-2 RNA	Non-structural polyprotein (pp1ab)	1.28×10^4 GE/mL	-PPA: 97%<br>-NPA: 100%	Approved (H)
P23 Labs, LLC.	P23 Labs TaqPath SARS-CoV-2 Assay	-OP, NP, AN and MT swabs<br>- Nasal and NP wash/aspirate<br>-BAL	- RT-PCR test for the qualitative detection of SARS-CoV-2 RNA<br>-The test is coupled with a self-collection kit	N, S and ORF1ab genes	10 copies/ µL	100%	Approved (H)
SolGent Co., Ltd.	DiaPlexQ™ Novel Coronavirus (2019-nCoV) Detection Kit	-OP, NP, AN and MT swabs<br>- Nasal and NP wash/aspirate<br>-BAL<br>-Sputum	- RT-PCR test for the qualitative detection of SARS-CoV-2 RNA<br>- Results in ~2 hours	ORF1a and N genes	200 copies/mL	100%	-Approved (H)<br>-CE marked<br>-EUA from Korean, Philippines authorities
BioCore Co., Ltd.	BioCore 2019-nCoV Real Time PCR Kit	-OP, NP, AN and MT swabs<br>- Nasal and NP wash/aspirate<br>-BAL<br>-Sputum and TA	- RT-PCR test for the qualitative detection of SARS-CoV-2 RNA	N and RdRp genes	500 copies/mL	100%	Approved (H)
Dba SpectronRx	Hymon SARS-CoV-2 Test Kit	-Nasal, OP, NP, AN and MT swabs	- RT-PCR test for the qualitative detection of SARS-CoV-2 RNA	N and E genes	5 copies/reaction	100%	Approved (H)

		-BAL					
Gravity Diagnostics, LLC	Gravity Diagnostics COVID-19 Assay	-Nasal, NP and OP swabs<br>-BAL	- RT-PCR test for the qualitative detection of SARS-CoV-2 RNA	N gene (N1 and N2 regions)	2.4 copies/µL	100%	Approved (H)
Phosphorus Diagnostics LLC	Phosphorus COVID-19 RT-qPCR Test	Saliva specimens	- RT-PCR test for the qualitative detection of SARS-CoV-2 RNA<br>-The test is coupled with a self-collection kit	N gene (N1 and N2 regions)	5 copies/µL	-PPA: 97.1%<br>-NPA: 96.5%	Approved (H)
Genetron Health (Beijing) Co., Ltd.	Genetron SARS-CoV-2 RNA Test	OP, NP, AN and MT swabs	- RT-PCR test for the qualitative detection of SARS-CoV-2 RNA	ORF1ab and N genes	1000 copies/mL	-PPA:100%<br>-NPA: 97.1%	Approved (H)
Euroimmun US Inc.	EURORealTime SARS-CoV-2	-MT, NP, and OP swabs<br>-BAL	- RT-PCR test for the qualitative detection of SARS-CoV-2 RNA	ORF1ab and N genes	150 copies/mL	PPA:96%<br>NPA: 100%	- Approved (H)<br>-CE marked<br>- Australian Therapeutic Goods Administration authorization
ChromaCode Inc.	HDPCR™ SARS-CoV-2 Assay	-NP, OP, AN and MT swabs<br>-Nasal aspirate/wash<br>-BAL	- RT-PCR test for the qualitative detection of SARS-CoV-2 RNA	N gene (N1 and N2 regions)	1000 copies/mL	QuantStudio 7system:<br>-PPA: 100%<br>-NPA: 96.7%<br>QuantStudio 12K Flex:<br>-PPA: 100%<br>-NPA: 96.7%	Approved (H)
Illumina Inc.	Illumina® COVIDSeq™ Test	-OP, NP, AN and MT swabs<br>- Nasal and NP wash/aspirate<br>-BAL	Next-Generation Sequencing (NGS) in vitro diagnostic test		1000 copies/mL	-PPA: 98.1%<br>-NPA: 97.4%	Approved (H)
TBG Biotechnology Corp	ExProbeTM SARS-CoV-2 Testing Kit	-OP, NP, AN and MT swabs<br>- Nasal and NP wash/aspirate<br>-BAL	- RT-PCR test for the qualitative detection of SARS-CoV-2 RNA	RdRp, N and E genes	10 copies/µL	-PPA: 100%<br>-NPA: 99.5%	Approved (H)
Tide Laboratories, LLC	DTPM COVID-19 RT-PCR test	NP, OP and MT swabs	- RT-PCR test for the qualitative detection of SARS-CoV-2 RNA	N gene	22 GE/ µL	100%	Approved (H)
Xiamen Zeesan Biotech Co., Ltd.	SARS-CoV-2 Test Kit (Real-time PCR)	-NP, OP, AN and MT swabs<br>-Nasal and NP wash/aspirate<br>-BAL	- RT-PCR test for the qualitative detection of SARS-CoV-2 RNA	ORF1 and N genes	200 copies/mL	100%	-Approved (H)<br>-CE marked
University of California San Diego Health	UCSD RC SARS-CoV-2 Assay	-Self-collected nasal swabs<br>-Clinician collected NP, OP, AN and MT swabs	- RT-PCR test for the qualitative detection of SARS-CoV-2 RNA<br>-The test is limited to University of California San Diego Health at Center for Advanced Laboratory Medicine	ORF1ab gene	N.A.	-PPA is >96%<br>-NPA is >96%	Approved (H)
Clinical Reference Laboratory, Inc.	CRL Rapid Response	Saliva specimens	- reverse RT-PCR test for the qualitative detection of SARS-CoV-2 RNA<br>-The test is coupled with a self-collection kit	RdRp gene	250 copies/mL	100%	Approved (H)
Eli Lilly and Company	Lilly SARS-CoV-2 Assay	-NP, OP, AN and MT swabs<br>-Nasal wash/aspirate<br>-BAL	- RT-PCR test for the qualitative detection of SARS-CoV-2 RNA	N gene (N1 and N2 regions)	10^3 copies/mL	-PPA: 100%<br>-NPA: 93.3%	Approved (H)

In-Vitro Diagnostics for Rapid and High-Accuracy COVID-19 Screening.

Sandia National Laboratories	SNL-NM 2019 nCoV Real-Time RT-PCR Diagnostic Assay	-NP, OP, AN and MT swabs<br>-Nasal wash/aspirate<br>-BAL	- RT-PCR test for the qualitative detection of SARS-CoV-2 RNA	N gene	6.25 copies/µL	100%	Approved (H)
Jiangsu CoWin Biotech Co., Ltd.	Novel Coronavirus (SARS-CoV-2) Fast Nucleic Acid Detection Kit (PCR-Fluorescence Probing)	OP swabs	- RT-PCR test for the qualitative detection of SARS-CoV-2 RNA	ORF1ab and N genes	300 copies/mL	100%	Approved (H)
Helix OpCo LLC (dba Helix)	Helix COVID-19 Test	NP, OP, AN and MT swabs	- RT-PCR test for the qualitative detection of SARS-CoV-2 RNA	N, S and ORF1ab genes	1000 genome copy equivalents/mL	-PPA: 96.7%<br>-NPA: 100%	Approved (H)
DiaCarta, Inc.	QuantiVirus SARS-CoV-2 Multiplex Test Kit	-Nasal, NP and OP swabs<br>-Sputum	- RT-PCR test for the qualitative detection of SARS-CoV-2 RNA<br>- Results in <2 hours	ORF1ab	-100 copies/mL in ABI QuantStudio 5 and Bio-Rad CFX 384<br>-50 copies/mL in ABI 7500 Fast Dx system	-PPA: 97.6%<br>-NPA: 100%	Approved (H)
Access Genetics, LLC	OraRisk COVID-19 RT-PCR	Nasal and NP swabs	- RT-PCR test for the qualitative detection of SARS-CoV-2 RNA	RdRp gene	15 viral copies/µL	NP swabs:<br>PPA and NPA:100%Nasal swabs:<br>-PPA: 100%<br>-NPA: 95.7%	Approved (H)
Boston Heart Diagnostics	Boston Heart COVID-19 RT-PCR Test	-NP, OP, MT and nasal swabs<br>-NP aspirate<br>-BAL	- RT-PCR test for the qualitative detection of SARS-CoV-2 RNA	N, S and ORF1ab genes	250 copies/mL	-PPA: 96.7%<br>-NPA: 96.7%	Approved (H)
KogeneBiotech Co., Ltd.	PowerChek 2019-nCoV Real-time PCR Kit	-NP, OP, AN and MT swabs<br>-Nasal and NP wash/aspirate<br>-BAL<br>-Sputum	- RT-PCR test for the qualitative detection of SARS-CoV-2 RNA	RdRp and E genes	4 copies/ µL	100%	Approved (H)
Trax Management Services Inc.	PhoenixDx SARS-CoV-2 Multiplex	-NP, OP, AN and MT swabs<br>-BAL	- RT-PCR test for the qualitative detection of SARS-CoV-2 RNA	ORF1ab and N genes	50 copies/ µL	100%	Approved (H)
Compass Laboratory Services, LLC	Compass Laboratory Services SARS-CoV2 Assay	-NP, OP, AN and MT swabs<br>-Nasal and NP wash/aspirate	-RT-PCR test for the qualitative detection of SARS-CoV-2 RNA	N.A.	5 copies/ µL	-PPA: 100%<br>-NPA: 96%	Approved (H)
Boston Medical Center	BMC-CReM COVID-19 Test	-NP, OP, AN and MT swabs<br>-Nasal and NP wash/aspirate<br>-BAL	-RT-PCR test for the qualitative detection of SARS-CoV-2 RNA	N gene (N1 and N2 regions)	1 gene copy/ µL	100%	Approved (H)
UCSF Health Clinical Laboratories, UCSF Clinical Labs at China Basin	SARS-CoV-2 RNA DETECTR Assay	-NP, OP, AN and MT swabs<br>-Nasal and NP wash/aspirate	-RT-PCR test for the qualitative detection of SARS-CoV-2 RNA	N gene	20 copies/ µL	-PPA: 95%<br>-NPA: 100%	Approved (H)
BioSewoom, Inc.	Real-Q 2019-nCoV Detection Kit	-NP, OP, nasal and MT swabs<br>-Tracheal and NP aspirate<br>-BAL	-RT-PCR test for the qualitative detection of SARS-CoV-2 RNA	RdRp and E genes	6.25 copies/ µL	100%	- Approved (H)<br>- CE marked
Clinical Research Sequencing Platform (CRSP), LLC at the Broad Institute of MIT and Harvard	CRSP SARS-CoV-2 Real-time Reverse Transcriptase (RT)-PCR Diagnostic Assay	-NP, OP, AN and MT swabs<br>-Nasal and NP wash/aspirate<br>-BAL	-RT-PCR test for the qualitative detection of SARS-CoV-2 RNA<br>-Testing is limited to the Clinical Research Sequencing Platform (CRSP), LLC at the Broad Institute of MIT and Harvard	N gene (N1 and N2 regions)	-4.8 x 10^3 copies/mL for Assay Version 1<br>-1.6 x 10^3 copies/mL for Assay Version 2	100%	Approved (H)

Enzo Life Sciences, Inc.	AMPIPROBE SARS-CoV-2 Test System	-NP, OP, AN and MT swabs<br>-Nasal and NP wash/aspirate	-Multiplexed RT-PCR test for the qualitative detection of SARS-CoV-2 RNA	N gene (N1 and N2 regions)	280 copies/mL	-PPA: 96.2%<br>-NPA: 98%	Approved (H)
Access Bio, Inc.	CareStart COVID-19 MDx RT-PCR	-NP, OP, nasal swabs<br>-Nasal and NP	-RT-PCR test for the qualitative detection of SARS-CoV-2 RNA	RdRp and N genes	N.A.	100%	Approved (H)
Gene By Gene	Gene By Gene SARS-CoV-2 Detection Test	NP and nasal swabs	-RT-PCR test for the qualitative detection of SARS-CoV-2 RNA	N gene (N1 and N2 regions)	6.25 copies/ µL	100%	Approved (H)
Laboratorio Clinico Toledo	Laboratorio Clinico Toledo SARS-CoV-2 Assay	-Nasal, NP, MT, OP, BAL swabs	-RT-PCR test for the qualitative detection of SARS-CoV-2 RNA	N and E genes	203cp/mL	100	Approved (H)
Centers for Disease Control and Prevention (CDC)	Influenza SARS-CoV-2 (Flu SC2) Multiplex Assay	-NP, OP, and nasal swabs.<br>-BAL, lower respiratory tract aspirates, and nasopharyngeal wash/aspirate or nasal aspirate.	- Multiplexed RT-PCR test for the simultaneous qualitative detection and differentiation of SARS-CoV-2, influenza A virus, and/or influenza B virus nucleic acid.	N gene	$10^{6.5}$ $TCID_{50}$/mL	N.A.	Approved (H)
CENTOGENE US, LLC	CentoFast-SARS-CoV-2 RT-PCR Assay	-Dry OP swabs	-RT-PCR test for the qualitative detection of SARS-CoV-2 RNA<br>-Testing is limited to CENTOGENE or other laboratories designated by CENTOGENE	E and RdRP genes.	5 viral copies/µL	100%	Approved (H)
Psomagen, Inc.	Psoma COVID-19 RT Test	-nasal, MT, NP, OP swabs<br>-BAL	-rRT-PCR test for the qualitative detection of SARS-CoV-2 RNA	N gene (N1 and N2 regions)	1 cp/µL	100%	Approved (H)
TNS Co., Ltd (Bio TNS	COVID-19 RT-PCR Peptide Nucleic Acid (PNA) kit	-NP, OP, AN, MT, BAL swabs<br>-nasopharyngeal wash/aspirates or nasal aspirates	-RT-PCR test for the qualitative detection of SARS-CoV-2 RNA	RdRP and N genes	3.715 copies/ µL for N gene<br>-2.524 copies/µL for RdRP gene	100%	Approved (H)
LifeHope Labs	LifeHope 2019-nCoV Real-Time RT-PCR Diagnostic Panel	-Nasal, MT, NP, and OP swabs<br>-BAL	-rRT-PCR test for the qualitative detection of SARS-CoV-2 RNA	N gene (N1 and N2 regions)	2.5 GE/µL	100%	Approved (H)
Acupath Laboratories, Inc	Acupath COVID-19 Real-Time (RT-PCR) Assay	-NP swab, NP aspirate, and BAL	-RT-PCR test for the qualitative detection of SARS-CoV-2 RNA	ORF1ab, N, and S genes	25 copies/ µL	100%	Approved (H)
Inform Diagnostics, Inc.	Inform Diagnostics SARS-CoV-2 RT-PCR Assay	-NP, OP, AN, MT swabs<br>-NP wash/aspirate or nasal aspirates<br>-BAL	-RT-PCR test for the qualitative detection of SARS-CoV-2 RNA	N gene (N1 and N2 regions)	20 copies/µL	-PPA: 93.94%<br>-NPA: 100%	Approved (H)
Diagnostic Solutions Laboratory, LLC	DSL COVID-19 Assay	-nasal, MT, NP, OP swabs<br>-BAL	-RT-PCR test for the qualitative detection of SARS-CoV-2 RNA	N (N1 and N3 regions) and S genes	10 cp/swab	-PPA: 100%<br>-NPA: 97%	Approved (H)
PreciGenome LLC	FastPlex Triplex SARS-CoV-2 detection kit (RT-Digital PCR	-OP swabs	-Digital RT-PCR test for the qualitative detection of SARS-CoV-2 RNA	ORF1ab and N genes	571.4 copies/mL	-PPA: 96.3% -NPA: 96.7%	Approved (H)
PlexBio Co., Ltd.	IntelliPlex SARS-CoV-2 Detection Kit	-NP, OP, AN, MT nasal swabs.<br>-nasopharyngeal	-RT-PCR test for the qualitative detection of SARS-CoV-2 RNA<br>- Used in combination with πCode technology and the IntelliPlex	RdRP, E, and N genes	140 copies/mL	N.A.	Approved (H)

		wash/aspirate or nasal aspirates<br>-BAL	1000 πCode Processor and PlexBio 100 Fluorescent Analyzer with DeXipher software				
University of Texas MD Anderson Cancer Center, Molecular Diagnostics Laboratory	MD Anderson High-throughput SARS-CoV-2 RT-PCR Assa	-NP, OP, MT, and nasal swabs.	-RT-PCR test for the qualitative detection of SARS-CoV-2 RNA<br>-Testing is limited to the Molecular Diagnostic Laboratory (MDL) at the University of Texas MD Anderson Cancer Center	N1 and N2 genes	5 copies/µL	100%	Approved (H)
HealthQuest Esoterics	HealthQuest Esoterics TaqPath SARS-CoV-2 Assay	-NP, OP, AN, MT nasal swabs.<br>- NP wash/aspirate or nasal aspirates<br>-BAL	-RT-PCR test for the qualitative detection of SARS-CoV-2 RNA	ORF1ab, N, and S genes	20 copies/µL	100%	Approved (H)
University of Alabama at Birmingham Fungal Reference Lab	FRL SARS CoV-2 Test	-nasal, NP, OP, MT, AN swabs<br>-nasopharyngeal wash/aspirate, nasal aspirate<br>-BAL	-RT-PCR test for the qualitative detection of SARS-CoV-2 RNA<br>-Testing is limited to the UAB Fungal Reference Lab in Birmingham, Alabama	N gene (N1 region)	125 copies/mL	100%	Approved (H)
Gencurix, Inc.	GenePro SARS-CoV-2 Test	-NP, OP, AN, and MT swabs<br>- NP wash/ aspirate, nasal aspirate<br>-BAL	-RT-PCR test for the qualitative detection of SARS-CoV-2 RNA	Orf1ab and E genes	840 GE/mL	100%	- Approved (H)<br>- CE marked<br>- Philippines FDA
Jiangsu Bioperfectus Technologies Co., Ltd.	COVID-19 Coronavirus Real Time PCR Kit	-NP, OP, AN, MT swabs<br>- nasal aspirates, nasal washes<br>-BAL and sputum	- Fluorescent RT-PCR test for the qualitative detection of SARS-CoV-2 RNA	ORF1ab and N genes	3.5×100 copies/mL	100%	Approved (H)
3B Blackbio Biotech India Ltd., a subsidiary of Kilpest India Ltd.	TRUPCR SARS-CoV-2 Kit	-NP, OP, AN, MT swabs<br>-NP aspirates/washes or nasal aspirates<br>-BAL	-RT-PCR test for the qualitative detection of SARS-CoV-2 RNA	RdRP and N genes	10 copies/µL	-PPA: 96.77%<br>-NPA: 96.67%	Approved (H)
The Ohio State University Wexner Medical Center	OSUWMC COVID-19 RT-PCR test	- Nasal, OP, NP swabs	-RT-PCR test for the qualitative detection of SARS-CoV-2 RNA<br>- Testing is limited to the Ohio State University Wexner Medical Center	N gene (N1 and N2 regions)	0.25 cp/µL	100%	Approved (H)
Omnipathology Solutions Medical Corporation	Omni COVID-19 Assay by RT-PCR	-NP, OP, AN, MT swab<br>-nasopharyngeal wash/aspirate, nasal aspirate<br>-BAL	-RT-PCR test for the qualitative detection of SARS-CoV-2 RNA<br>-Testing is limited to Omnipathology Solutions Medical Corporation in Pasadena, California	N gene (N1 and N2 regions)	1.23 copies/µL	-PPA: 96.7%<br>-NPA: 100%	Approved (H)
Applied BioCode, Inc.	BioCode SARS-CoV-2 Assay	-NP, OP, and nasal swabs<br>-BAL	-RT-PCR test for the qualitative detection of SARS-CoV-2 RNA<br>-End-point detection of amplified DNA	-N gene	1.72 x10^{-2} $TCID_{50}$/mL.	100%	Approved (H)

			sequences is coupled to barcoded magnetic beads (BMB)				
RTA Laboratories Biological Products Pharmaceutical and Machinery Industry	Diagnovital SARS-CoV-2 Real-Time PCR Kit	-AN, MT, NP, OP<br>-nasopharyngeal wash/aspirates or nasal aspirates<br>-BAL	-RT-PCR test for the qualitative detection of SARS-CoV-2 RNA	-E and RdRP genes	38 copies/mL	100%	Approved (H)
**Isothermal Amplification Assays for SARS-CoV-2 Detection**							
Atila BioSystems, Inc.	iAMP® COVID-19 Detection Kit	NP and OP swab specimen	-Real-time fluorescent isothermal PCR assay for the qualitative detection of SARS-CoV-2 RNA<br>- results in <1.5 hours	ORF1ab and N genes	10 copies/µL	-PPA: 96.3%<br>-NPA: 97%	roved (H)
Abbott Diagnostics Scarborough, Inc.	Abbott ID NOW COVID-19	nasal, NP or throat swabs	- Real-time fluorescent isothermal PCR assay for the qualitative detection of SARS-CoV-2 RNA<br>-Result read: 5 minutes for positive results and 13 minutes for negative results	RdRp gene	125 GE/mL	100%	Approved (H)
SEASUN BIOMATERIALS Inc	AQ-TOP™ COVID-19 Rapid Detection Kit	-OP and NP swab<br>- AN and MT nasal swabs,<br>-NP wash/aspirate or nasal aspirate<br>-BAL and sputum	-Real-Time Loop-Mediated Isothermal Amplification (RT-LAMP) test intended for the qualitative detection of nucleic acid from SARS-CoV-2	ORF1ab	7 copies/ µL	100%	-Approved (H)<br>-CE marked
Cue Health Inc.	Cue COVID-19 Test	Nasal swabs	-An isothermal nucleic acid amplification assay intended for the qualitative detection of nucleic acid from the SARS-CoV-2	N gene	N.A.	100%	Approved (H)
**CRISPR-based Assays for SARS-CoV-2 Detection**							
Sherlock BioSciences, Inc.	Sherlock CRISPR SARS-CoV-2 Kit	-Nasal, NP, OP swabs<br>-NP wash/aspirate or nasal aspirate<br>-BAL	-CRISPR-cas based test for qualitative detection of SARS-CoV-2 RNA<br>-Combines Cas 13 enzyme with Cepheid's GeneXpert test processing<br>-RT-LAMP amplification followed by active collateral cleavage activity of Cas13a enzyme. The fluorescent readout is detected by a plate reader.	ORF1ab and N gene	-6.75 copies/µL for ORF1ab gene<br>-1.35 copies/µL for N gene	100%	Approved (H)
Mammoth Biosciences, Inc.	SARS-CoV-2 DETECTR Reagent Kit	-NP, OP, MT, AN swabs<br>-NP wash/aspirate or nasal aspirate	-CRISPR-cas based test for qualitative detection of SARS-CoV-2 RNA<br>-RT-LAMP amplification followed by active collateral	RdRp and N genes	20 copies/µL	-PPA: 95%<br>-NPA: 100%	Approved (H)

			cleavage activity of Cas12a enzyme. The fluorescent readout is detected by a plate reader.<br>-Results are obtained in 30-40 mins				
**Rapid Lateral Flow Immunoassays for Antibodies Detection**							
Autobio Diagnostics Co. Ltd.	Anti-SARS-CoV-2 Rapid Test	Human plasma or serum	-lateral flow immunoassay intended for the qualitative detection and differentiation of IgM and IgG antibodies to SARS-CoV-2<br>- results read after 15-20 min	IgM and IgG antibodies	5 µL of the serum or plasma sample	-PPA: 88.15% in 405 RT-PCR positive samples<br>-NPA: 99.04% in 312 RT-PCR negative samples	pproved (H)<br>- CE marked
Cellex Inc.	qSARS-CoV-2 IgG/IgM Rapid Test	Serum, plasma (EDTA, citrate) or venipuncture whole blood specimens	-lateral flow immunoassay intended for the qualitative detection and differentiation of IgM and IgG antibodies to SARS-CoV-2<br>- results read after 15-20 min	IgM and IgG antibodies	10µL of the specimen	-PPA: 88.2% in 128 RT-PCR positive samples<br>-NPA: 92.8% in 250 RT-PCR negative samples	pproved (H)<br>- CE marked<br>- Brazilian Health Regulatory Agency authorization<br>- Australian Therapeutic Goods Administration authorization
Healgen Scientific LLC	COVID-19 IgG/IgM Rapid Test Cassette (Whole Blood/Serum/Plasma)	Human venous whole blood, plasma from anticoagulated blood (Li+ heparin, K2EDTA and sodium citrate), or serum.	-A lateral flow immunoassay intended for the qualitative detection and differentiation of IgM and IgG antibodies to SARS-CoV-2	IgM and IgG antibodies	5 µL	For IgM:<br>-PPA: 86.7%<br>-NPA: 99%<br>For IgG:<br>-PPA: 96.7%<br>-NPA: 98%	Approved (H, M)
Hangzhou Biotest Biotech Co., Ltd.	RightSignTM COVID-19 IgG/IgM Rapid Test Cassette	Human venous whole blood (sodium heparin, EDTA, and sodium citrate), serum or plasma (sodium heparin, potassium EDTA and sodium citrate)	-A rapid lateral flow chromatographic immunoassay intended for the qualitative detection and differentiation of IgM and IgG antibodies to SARS-CoV-2	IgM and IgG antibodies	10 µL	For IgM: PPA and NPA is 100%<br>For IgG:<br>-PPA: 93.3%<br>-NPA: 100%	Approved (H, M)
Xiamen Biotime Biotechnology Co., Ltd.	BIOTIME SARS-CoV-2 IgG/IgM Rapid Qualitative Test	Human serum, plasma and venous whole blood	Lateral flow immunoassay intended for qualitative detection and differentiation of IgM and IgG antibodies to SARS-CoV-2	IgM and IgG antibodies	10-15 µL	-PPA: 100% (>15 days post symptoms onset)<br>-NPA: 98.46% in serum samples and 100% in plasma samples	Approved (H, M)
Access Bio, Inc.	CareStart COVID-19 IgM/IgG	Human serum, plasma and venous whole blood	Immunochromatographic lateral flow assay for the qualitative detection and differentiation of IgM and IgG antibodies to SARS-CoV-2	IgM and IgG antibodies against N and S1 RBD proteins	10 µL	-PPA: 98.44%<br>-NPA: 98.90%	Approved (H, M)
Megna Health, Inc.	Rapid COVID-19 IgM/IgG Combo Test Kit	Human serum and plasma	Lateral flow immunoassay for qualitative detection	IgM and IgG antibodies against N protein	2 µL	-PPA: 90.48%<br>-NPA: 98.95%	Approved (H, M)

			and differentiation of IgM and IgG antibodies to SARS-CoV-2				
Salofa Oy	Sienna-Clarity COVIBLOCK COVID-19 IgG/IgM Rapid Test Cassette	Human serum, plasma and venous whole blood	Membrane-based lateral flow immunoassay for the qualitative detection and differentiation of IgG and IgM antibodies to SARS-CoV-2	IgM and IgG antibodies against S1 RBD protein	10 µL	-Combined IgM and IgG PPA: 89.66% in 29 positive samples collected 8-14 days post symptoms onset<br>-Combined IgM and IgG NPA: >89% in samples collected before COIV-19	Approved (H, M)
Beijing Wantai Biological Pharmacy Enterprise Co., Ltd.	WANTAI SARS-CoV-2 Ab Rapid Test	Human serum, plasma and venous whole blood	Lateral flow assay for the qualitative detection of total antibodies (including IgG and IgM) to SARS-CoV-2	Total antibodies against S1 RBD protein	10 µL	-PPA: 94.70%<br>-NPA: 98.89%	Approved (H, M)
Assure Tech. (Hangzhou Co., Ltd)	Assure COVID-19 IgG/IgM Rapid Test Device	Human serum, plasma and venous whole blood	Rapid lateral flow chromatographic immunoassay for the qualitative detection and differentiation of IgM and IgG antibodies to SARS-CoV-2	IgM and IgG antibodies against N and S1 proteins	5 µL	For IgM:<br>-PPA: 86.7% in 25 positive serum samples collected 8-14 days post symptoms onset, and 100% in 3 positive whole blood samples collected 8-14 days post symptoms onset<br>-NPA: 98.1%<br>For IgG:<br>-PPA: 86.7% in 15 positive serum samples collected 8-14 days post symptoms onset, and 100% in 3 positive whole blood samples collected 8-14 days post symptoms onset<br>-NPA: 100%	- Approved (H, M)<br>- CE marked<br>- Brazilian Health Regulatory Agency authorization
**Enzyme Linked Immunoassays for Antibodies Detection**							
EUROIMMUN US Inc.	Anti-SARS-CoV-2 ELISA (IgG)	Human serum or plasma (K+-EDTA, Li+-heparin, Na+-citrate) specimens	-Enzyme-Linked Immunosorbent Assays (ELISA) for the qualitative detection of S1 IgG antibodies to SARS-CoV-2	S1 IgG antibodies	10 µL serum in 1.0 mL sample buffer	-PPA: 90% in RT-PCR positive samples<br>-NPA: 100 % in PCR negative samples	-Approved (H)<br>-CE marked<br>- Brazilian Health Regulatory Agency authorization<br>-Australian Therapeutic Goods Administration authorization
Roche Diagnostics	Elecsys Anti-SARS-CoV-2	Human serum and plasma (K2EDTA, K3EDTA, Liheparin) specimens	-Electrochemiluminescence Immunoassay (ECLIA) intended for qualitative detection of antibodies to SARSCoV2<br>-Total duration of sandwich assembly: 18 minutes	Total antibodies against N protein in SARS-CoV-2	30 µL of sample (cobas e 411, cobas e 601, and cobas e 602 analyzers) or 18 µL of sample (cobas e 801 analyzer) are incubated with a biotinylated monoclonal IL-	-PPA: 99.81 % in 5272 RT-PCR positive samples	- Approved (H, M)<br>- CE Marked<br>- Australian Therapeutic Goods Administration authorization<br>- Singapore Health Sciences Authority<br>- Health Canada authorization

					6-specific antibody.		- Brazilian Health Regulatory Agency authorization
Bio-Rad Laboratories, Inc.	Platelia SARS-CoV-2 Total Ab assay	Human serum and plasma EDTA specimens	-Enzyme-Linked Immunosorbent Assay (ELISA) intended for the qualitative detection of total antibodies (IgM, IgA and IgG) to SARS-CoV-2	Total antibodies against N protein	100 µL	-PPA: 92.16% in 51 RT-PCR positive samples<br>-NPA: 99.56% in 687 RT-PCR negative samples	- Approved (H)<br>- CE marked<br>- Australian Therapeutic Goods Administration authorization<br>- Singapore Health Sciences Authority
Abbott Laboratories Inc.	SARS-CoV-2 IgG assay	Human serum and plasma specimens	- Chemiluminescent microparticle immunoassay (CMIA) intended for the qualitative detection of IgG antibodies to SARS-CoV-2	IgG antibodies	75 µL	-PPA: 96.77% in samples collected ≥14 days post-symptom onset<br>-PPA: 86.36% in samples collected 8-13 days post-symptom onset<br>-NPA: 99.63%	- Approved (H, M)<br>- CE marked
DiaSorin Inc.	LIAISON SARS-CoV-2 S1/S2 IgG	Human serum, and plasma specimens	-Chemiluminescent immunoassay (CLIA) for qualitative detection of S1 and S2 IgG antibodies to SARS-CoV-2	IgG antibodies	20 µL	-PPA: 97.65% in RT-PCR positive sampled collected at ≥15 days post-diagnosis<br>-PPA: 89.80% in RT-PCR positive sampled collected at 6-14 days post-diagnosis<br>-NPA: 99.3% (108/1090 presumed negative samples)	- Approved (H, M)<br>-CE marked<br>- Health Canada authorization<br>- Brazilian Health Regulatory Agency authorization
Ortho-Clinical Diagnostics, Inc.	VITROS Immunodiagnostic Products Anti-SARS-CoV-2 IgG Reagent Pack	Human serum specimens	-Chemiluminescent immunoassay (CLIA) test for the qualitative detection of IgG antibodies to SARS-CoV-2<br>-time to first result is 48 min	IgG antibodies against S protein	20 µL	-PPA: 87.5% in 48 RT-PCR positive samples<br>-NPA: 100% in 407 presumed negative samples	- Approved (H, M)<br>- CE marked
Mount Sinai Laboratory	COVID-19 ELISA IgG Antibody Test	Human serum and plasma specimens	-Enzyme-Linked Immunosorbent Assays (ELISA) for the qualitative detection of human Sand RBD IgG antibodies to SARS-CoV-2	IgG antibodies against RBD protein	N.A.	-PPA: 92% in RT-PCR positive samples<br>-NPA: 100% in RT-PCR negative samples	Approved (H)
Siemens Healthcare Diagnostics Inc.	ADVIA Centaur® SARS-CoV-2 Total (COV2T) assay	Human serum and plama (potassium EDTA and lithium heparin) specimens	-Chemiluminescent immunoassay (CLIA) intended for qualitative and semi-quantitative detection of IgG antibodies to SARS-CoV-2	IgG antibodies	10 µL	-PPA: 93.44% in 61 RT-PCR positive samples by collected at 7-13 days post-testing<br>-NPA: 99.88% in 1734 presumed negative samples	Approved (H, M)

						(healthy individuals)	
Siemens Healthcare Diagnostics Inc.	Atellica® IM SARS-CoV-2 Total (COV2T) assay	Human serum and plasma (potassium EDTA and lithium heparin) specimens	-Chemiluminescent immunoassay (CLIA) intended for qualitative detection of total antibodies (IgG and IgM) to SARS-CoV-2	IgG and IgM antibodies against S1 protein	10 µL	-PPA: 92.19% in 64 RT-PCR positive samples by collected 7-13 post-testing<br>-NPA: 99.95% in 1841 presumed negative samples (healthy individuals)	- Approved (H, M)<br>- CE Marked<br>- Australian Therapeutic Goods Administration authorization<br>- Singapore Health Sciences Authority<br>- Health Canada authorization
Vibrant America Clinical Labs	Vibrant COVID-19 Ab assay	Human serum or Dry Blood Spot (DBS) using fingerstick blood specimen	-Chemiluminescence immunoassay (CLIA) intended for the qualitative detection and differentiation of IgM and IgG antibodies to SARS-CoV-2	IgM and IgG against S1 subunit, RBD, S2 subunit and N proteins	N.A.	-PPA: 98.11% in 59 presumed positive samples<br>-NPA: 98.6% in 495 presumed negative samples (healthy individuals)	Approved (H)
Siemens Healthcare Diagnostics Inc.	Dimension® EXL™ SARS-CoV-2 Total antibody assay (CV2T)	Human serum and plasma (dipotassium EDTA and lithium heparin) specimens	-Chemiluminescent immunoassay (CLIA) intended for the qualitative detection of total antibodies (including IgG and IgM) to SARS-CoV-2	IgG and IgM against S1 proteins	10 µL	-PPA: 77.37% in 38 RT-PCR positive samples by collected 7-13 post-testing<br>-NPA: 99.87% in 1529 presumed negative samples (healthy individuals)	Approved (H, M)
Siemens Healthcare Diagnostics Inc.	Dimension Vista® SARS-CoV-2 Total antibody assay (COV2T)	Human serum and plasma (dipotassium EDTA and lithium heparin)	-Chemiluminescent immunoassay (CLIA) intended for qualitative detection of total antibodies (IgG and IgM) antibodies to SARS-CoV-2	IgG and IgM against S1 proteins	10 µL	-PPA: 97.37% in 38 RT-PCR positive samples by collected 7-14 post-testing<br>-NPA: 99.8% in 1529 presumed negative samples (healthy individuals)	Approved (H, M)
InBios International, Inc.	SCoV-2 Detect™ IgG ELISA	Human serum specimens	-Enzyme-Linked Immunosorbent Assays (ELISA) for the qualitative detection of IgG antibodies to SARS-CoV-2<br>-up to 90 specimens can be evaluated with each kit<br>-The entire assay takes around 1 hour	IgG antibodies	N.A.	-PPA: 100% in RT-PCR positive samples<br>-PPA: 100% in RT-PCR positive samples collected 8-14 days post symptoms onset<br>-NPA: 98.95% in 94 presumed negative specimens (healthy individuals)	Approved (H)
Beckman Coulter, Inc.	Access SARS-CoV-2 IgG	Human serum, serum separator tubes, and plasma (lithium heparin, dipotassium EDTA, tripotassium EDTA, and sodium citrate)	-Paramagnetic particle chemiluminescent immunoassay (CLIA) intended for the qualitative detection of IgG antibodies to SARS-CoV-2	IgG antibodies against RBD of the S1 protein	N.A.	-PPA: 95.3% in presumed RT-PCR positive samples collected 4-14 days post testing<br>-NPA>99%	- Approved (H, M)<br>- CE marked
Babson Diagnostics, Inc.	Babson Diagnostics aC19G1	Human serum and plasma (potassium EDTA,	- Indirect sandwich chemiluminescent immunoassay (CLIA) performed on the	IgG antibodies	N.A.	-PPA: 60.96% in RT-PCR positive EDTA plasma samples collected	Approved (H)

		lithium heparin)	Atellica IM Analyzer intended for the qualitative detection of IgG antibodies to SARS-CoV-2			8-14 days post testing<br>-PPA: 32.57% in positive serum samples collected 8-14 days post testing<br>-NPA: 100%	
Hangzhou Laihe Biotech Co., Ltd.	LYHER Novel Coronavirus (2019-nCoV) IgM/IgG Antibody Combo Test Kit (Colloidal Gold)	Serum and plasma (dipotassium-EDTA, lithium-heparin, or sodium-citrate)	-Lateral flow immunoassay intended for the qualitative detection of and differentiation of IgG and IgM antibodies to SARS-CoV-2<br>- time-to-results is 10-15 minutes	IgG and IgM antibodies against S protein	50 µL	For IgM:<br>-PPA: 85.71% from 21 RT-PCR positive samples collected 7-14 days post symptoms onset<br>-NPA: 99.43%<br>For IgG:<br>-PPA: 76.19% from 21 RT-PCR positive samples collected 7-14 days post symptoms onset<br>-NPA: 99.43%	-Approved (H, M)<br>-CE marked<br>- Brazilian Health Regulatory Agency authorization<br>- Australian Therapeutics Administration authorization
Biohit Healthcare (Hefei) Co. Ltd.	Biohit SARS-CoV-2 IgM/IgG Antibody Test Kit	Human serum, plasma (heparin, dipotassium EDTA, and sodium citrate), and venous whole blood (heparin, dipotassium EDTA, and sodium citrate).	-Rapid immunochromatographic test for the qualitative detection and differentiation of IgM and IgG antibodies to SARS-CoV-2<br>- time-to-results is 15 minutes	IgG and IgM antibodies against N protein	10 µL	For IgM:<br>-PPA: 83.02% in presumed positive samples 4-14 days post symptoms onset<br>-NPA: 99.46%<br>For IgG:<br>-PPA: 56.6% in RT-PCR positive samples collected 4-14 days post symptoms onset<br>-NPA: 100%	- Approved (H, M)<br>- CE marked
Emory Medical Laboratories	SARS-CoV-2 RBD IgG test	Human serum	Enzyme-Linked Immunosorbent Assay (ELISA) intended for the qualitative detection of IgG antibodies to SARS-CoV-2	IgG antibodies against RBD fusion protein	N.A.	- PPA: 100% in 38 presumed positive samples collected 8-14 days post testing<br>-NPA: 97.68%	Approved (H)
Wadsworth Center, New York State Department of Health	New York SARS-CoV Microsphere Immunoassay for Antibody Detection	Human serum specimens	Microsphere Immunoassay (MIA) intended for qualitative detection of total antibodies (IgG, IgM and IgA) to SARSCoV2	Total antibody against N protein	N.A.	-PPA: 79.3% in 334 PCR positive samples collected 20 days post symptom onset<br>-NPA: >96% in samples collected from different set of sera	Approved (H)
Siemens Healthcare Diagnostics Inc.	ADVIA Centaur SARS-CoV-2 IgG (COV2G)	Human serum and plasma	Chemiluminescent immunoassay for qualitative and semi-quantitative detection of IgG antibodies to SARS-CoV-2	IgG antibodies	10 µL	-PPA: 100% in RT-PCR positive samples collected >14 days posttesting<br>-NPA: 99.89%	Approved (H, M)
Luminex Corporation	xMAP SARS-CoV-2 Multi-Antigen IgG Assay	Human serum and plasma	Multiplex, microsphere-based assay for qualitative detection of IgG antibodies to SARS-CoV-2	IgG antibodies against N, RBD and S1 proteins	10 µL	-PPA: 100%<br>-NPA: 99.2%	Approved (H)
Diazyme Laboratories, Inc.	Diazyme DZ-Lite SARS-CoV-2 IgG CLIA Kit	Human serum and plasma	Chemiluminescent immunoassay for the qualitative detection of IgG antibodies to SARS-CoV-2	IgG antibodies	10 µL	-PPA: 100% in RT-PCR positive samples collected >15 days post testing<br>-NPA: 97.4%	Approved (H, M)

InBios International, Inc.	SCoV-2 Detect IgM ELISA	Human serum	Enzyme-Linked Immunosorbent Assay (ELISA) for the qualitative detection of IgM antibodies to SARS-CoV-2	IgM antibodies	4 µL	-PPA: 92.50%<br>-NPA: 98.95%	Approved (H)
**Antigen-based Assays for SARS-CoV-2 Detection**							
Quidel Corporation	Sofia 2 SARS Antigen FIA	Direct NP and nasal swab specimens	- Lateral flow immunofluorescent sandwich assay for the qualitative detection of the N protein antigen in SARS-CoV-2<br>- time-to-results is 15 minutes	N protein antigen	$1.13x10^2$ $TCID_{50}$/mL	- PPA: 80% in 59 RT-PCR positive frozen NP samples<br>- NPA: 100% in 84 RT-PCR negative frozen NP samples	-Approved (H, M)<br>-Authorized for distribution and use in patient care settings under a CLIA Certificate of Waiver (Certificate of Compliance)
Becton, Dickinson and Company (BD)	BD Veritor System for Rapid Detection of SARS-CoV-2	Direct nasal swab specimens	-Chromatographic digital immunoassay intended for the direct and qualitative detection the N protein antigen in SARS-CoV-2<br>- time-to-results is 15 minutes	N protein antigen	1.4 x 102 TCID50/mL	-PPA: 84% in RT-PCR positive samples<br>-NPA: 100% in RT-PCR negative samples	-Approved (H, M)<br>-Authorized for distribution and use in patient care settings under a CLIA Certificate of Waiver (Certificate of Compliance)
LumiraDx UK Ltd.	LumiraDx SARS-CoV-2 Ag Test	Direct nasal swab specimens	-Rapid microfluidic immunofluorescence assay for qualitative detection of the N protein antigen in SARS-CoV-2<br>- time-to-results is 12 minutes	N protein antigen	$2.8 x 10^5$ $TCID_{50}$/m	-PPA is 97.6%<br>-NPA is 96.6%	-Approved (H, M)<br>-Authorized for distribution and use in patient care settings under a CLIA Certificate of Waiver (Certificate of Compliance)
Abbott Diagnostics Scarborough, Inc.	BinaxNOW COVID-19 Ag Card	Direct nasal swab specimens	- Lateral flow immunofluorescent sandwich assay for the qualitative detection of the N protein antigen in SARS-CoV-2<br>- time-to-results is 15 minutes	N protein antigen	22.5 $TCID_{50}$/swab	-PPA is 97.1% in presumed positive samples<br>-NPA is 98.5% in presumed negative samples	-Approved (H, M)<br>-Authorized for distribution and use in patient care settings under a CLIA Certificate of Waiver (Certificate of Compliance)
